# Suppression of inflammatory arthritis in human serum paraoxonase 1 transgenic mice

**DOI:** 10.1038/s41598-020-74016-w

**Published:** 2020-10-08

**Authors:** Christina Charles-Schoeman, Jennifer Wang, Ani Shahbazian, Yuen Yin Lee, Xiaoyan Wang, Victor Grijalva, Ernest Brahn, Diana M. Shih, Asokan Devarajan, Christy Montano, Aldons J. Lusis, Srinivasa T. Reddy

**Affiliations:** 1 Division of Rheumatology, Department of Medicine, University of California, 1000 Veteran Ave, Rm 32-59, Los Angeles, CA 90095 USA; 2 Department of Medicine Statistics Core, University of California, Los Angeles, Los Angeles, CA 90095 USA; 3 Division of Cardiology, Department of Medicine, University of California, Los Angeles, Los Angeles, CA 90095 USA; 4 UCLA Cardiac Arrhythmia Center, Neurocardiology Research Center of Excellence, Los Angeles, CA USA; 5 Departments of Human Genetics, Microbiology, Immunology, and Molecular Genetics, University of California, Los Angeles, CA 90095 USA

**Keywords:** Cardiology, Rheumatology

## Abstract

Paraoxonase 1(PON1) is an HDL-associated protein, which metabolizes inflammatory, oxidized lipids associated with atherosclerotic plaque development. Because oxidized lipid mediators have also been implicated in the pathogenesis of rheumatoid arthritis (RA), we evaluated the role of PON1 in murine inflammatory arthritis. K/BxN serum transfer (STIA) or collagen antibody transfer (CAIA) was used for arthritis induction in B6 mice homozygous for the PON1 human transgene [PON1Tg], PON1 knock-out mice [PON1KO], and wild type littermate control mice [WT]. Experiments were also performed in K/BxN mice with chronic arthritis, and in RA patients and healthy controls. Arthritis activity in K/BxN mice was associated with a marked dyslipidemia, lower PON1 activity and higher bioactive lipid mediators (BLM), as well as a dysregulated hepatic lipid gene expression profile. Higher serum PON1 activity correlated with lower BLM and lower arthritis activity in both K/BxN mice and RA patients. Overexpression of the human PON1 transgene was associated with reduced inflammatory arthritis, which correlated strongly with higher circulating PON1 activity, upregulation of the hepatic glutathione pathway, and reduction of circulating BLM. These results implicate PON1 as a potential novel therapeutic target for joint disease in RA with potential for vascular benefit, which warrants further investigation.

## Introduction

Rheumatoid arthritis (RA) is a chronic inflammatory disease of the joints associated with marked disability and high mortality due to cardiovascular (CV) disease^1^. While the emergence of multiple biologic therapies and small molecule inhibitors for RA has greatly improved outcomes in recent years, a large percentage of RA patients fail to reach remission despite these therapeutics, and daily chronic pain and disability persists^2^. Atherosclerotic vascular disease remains a leading cause of morbidity and mortality in RA patients^3^.

Lipid peroxidation products generated from oxidative stress are implicated in the development of both atherosclerosis and RA^4^. We previously reported high levels of oxidized fatty acids in dysfunctional high-density lipoproteins (HDL) from both synovial fluid and circulation of patients with active RA compared to controls^5^ . Dysfunctional, pro-inflammatory HDL has been associated with both atherosclerotic risk and disease activity in patients with rheumatic disease^6,7^.

Paraoxonase 1 (PON1) is a 45-kDa glycoprotein synthesized and highly expressed in the liver, which is closely associated with HDL in circulation and linked to HDL’s anti-oxidant capacity^8^. PON1 inhibits lipid peroxidation, hydrolyzes multiple bioactive lipid mediators (BLM) including oxidized phospholipids and eicosanoids, and prevents oxidation of low density lipoproteins (LDL) and HDL^9–11^. Higher PON1 activity has been associated with reduced CV risk in patients with and without RA^12,13^, and PON1 overexpression decreases atherosclerosis in animal models^14^. No work to date has examined the role of PON1 in murine inflammatory arthritis.

In the current study, we first describe a marked dyslipidemia involving abnormalities in hepatic lipid metabolism, suppression of circulating PON1 activity and high BLM in the K/BxN mouse model of RA. We show that suppressed PON1 activity associates with higher BLM in both K/BxN mice and humans with active RA. We then demonstrate that overexpression of the human PON1 transgene (Tg) improves the dyslipidemia in arthritic mice, preventing increases in BLM with arthritis induction and decreasing arthritis activity and damage. The hepatic glutathione pathway was markedly upregulated in PON1Tg mice compared to controls after arthritis induction and correlated with decreases in circulating BLM. We propose that the PON1 pathway may represent a novel therapeutic target for treatment of inflammatory arthritis in humans with RA with potential for vascular risk reduction, warranting further investigation.

## Results

### Inflammatory arthritis in K/BxN Mice is associated with dyslipidemia

**Figure 1:**
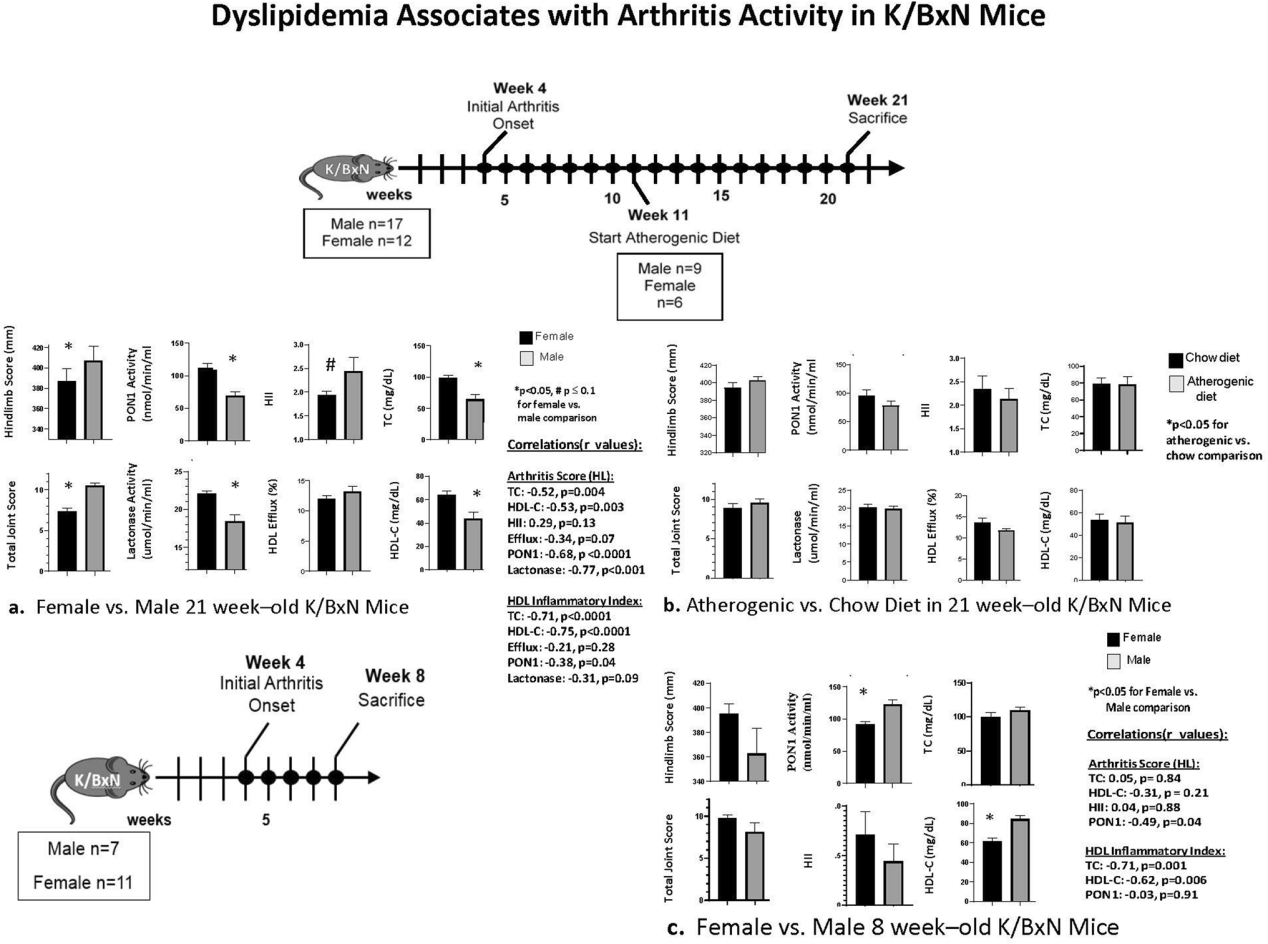
Associations of arthritis activity with dyslipidemia are shown in 21 week-old K/BxN mice (old, n = 29; 17 male, 12 female; (**A**,**B**)) and 8 week-old K/BxN mice (young, n = 18; 7 male, 11 female; (**C**)), which were generated by cross of NOD-Ag7 females and KRN males. In data shown in panels A and B, mice were maintained on chow until 11 weeks at which time 15/29 mice (6 female, 9 male) were switched to an atherogenic diet until sacrifice at 21 weeks. The remaining 14/29 mice were maintained on a chow diet until 21 weeks. (**A**) Comparisons of joint scores and lipid measures between more arthritic males with less arthritic females as well as correlations of arthritic scores with lipid measures. Correlations are also shown of HDL’s anti-oxidant capacity measured by the HDL inflammatory index (HII) with other lipid measures. (**B**) Comparisons of joint scores and lipid measures between mice on atherogenic versus chow diets. (**C**) Young mice were maintained on a standard mouse chow diet until sacrifice at 8 weeks. Comparisons of joint scores and lipid measures between the more arthritic females versus less arthritic males are shown as well as correlations of arthritic scores with lipid measures. Correlations are also shown of HDL’s anti-oxidant capacity measured by the HII with other lipid measures. Group comparisons and correlations were performed as described in the statistical analysis section of the “Materials and methods”*.* Data represent Mean ± SEM in the graphs.

Arthritis activity in K/BxN mice at 21 weeks was associated with reduction in serum PON1 activity and impaired HDL function. Male K/BxN mice had higher hind limb caliper measurements and total clinical arthritic scores compared to female littermates, which were associated with significantly lower PON1 activity (Fig. 1A). In addition, modest associations were noted between higher arthritic scores and reduction in HDL function measured by decreased efflux capacity as well as impairment in HDL’s anti-oxidant function (higher HII) (Fig. 1A).

K/BxN arthritis at 21 weeks was also associated with reductions in total and HDL cholesterol (HDL-C) levels, similar to reports in active RA patients^15^ More arthritic males had lower cholesterol levels compared to less arthritic females, and higher arthritic scores correlated significantly with lower cholesterol levels (Fig. 1A). Lower total and HDL-C cholesterol levels also correlated with worse HDL function as shown by a higher HII. In contrast, an atherogenic diet did not associate with differences in lipid levels or lipoprotein function. Mice on an atherogenic diet had no differences in cholesterol levels, PON1 activity, or HDL function compared to mice on a chow diet (Fig. 1B).

Associations of lipid measures with arthritic disease were also examined in young, 8 week-old arthritic K/BxN mice. In this experiment, female mice showed trends for higher arthritic scores compared to males and had significantly lower PON1 activity and HDL-C levels compared to males. Similar to the 21 week experiments, higher arthritic scores correlated with lower PON1 activity and HDL-C levels. In addition, lower total and HDL-C levels correlated with worse HDL function measured by a higher HII (Fig. 1C).

### Dyslipidemia in K/BxN mice associates with an abnormal cytokine and chemokine profile

The use of biologic and small molecule inhibitors of cytokine/chemokine pathways has been associated with increases in serum cholesterol levels in RA patients^16^. Because atherosclerotic risk is elevated in RA, increases in cholesterol with RA therapies raise safety concerns; the mechanisms for these cholesterol increases are not well understood. In the current work, we investigated associations of cytokine and chemokine levels with lipid measurements in 21 week-old K/BxN mice.

**Figure 2:**
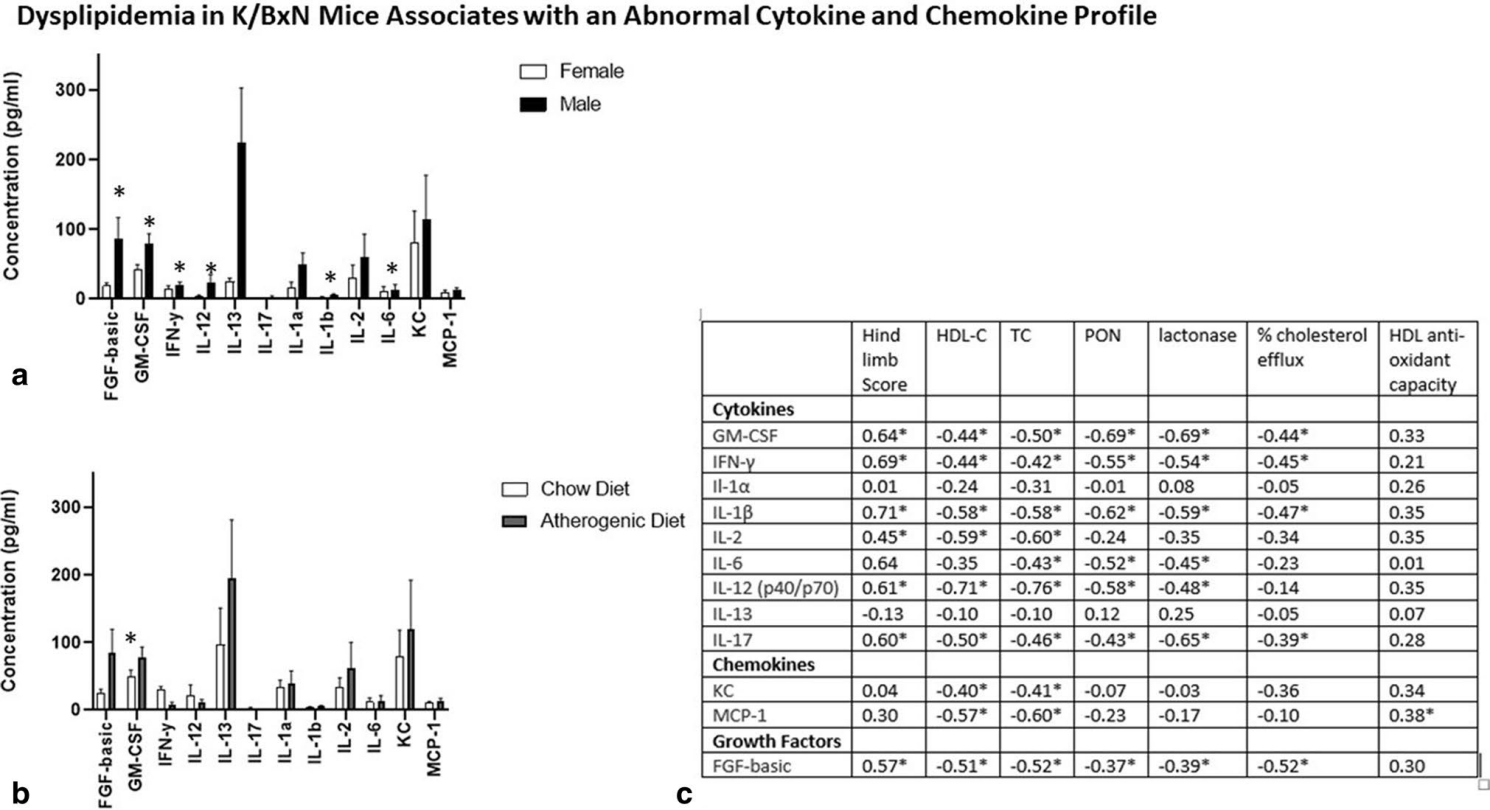
Associations of serum cytokine/chemokine levels with lipid measures, arthritis activity, and diet in 21 week-old K/BxN mice are shown. Serum cytokine/chemokine levels were assessed using Luminex-based 20 plex assays for the 29 21 week-old K/BxN mice from Fig. 1. (**A**) Cytokine/chemokine profiles are generally greater in the more arthritic males compared to less arthritic females. (**B**) No significant cytokine/chemokine differences were noted between mice on a chow versus atherogenic diet with the exception of G-CSF, which was higher in the mice fed an atherogenic diet. (**C**) Correlations of cytokine/chemokine levels with arthritic hindlimb scores and laboratory assays are shown. Of note, IL-4, IL-5, IL-10, TNF-α, IP-10, MIG, MIP-1α, and VEGF were assessed in the Luminex panel but values were too low in majority of specimens to allow reliable analysis. Hindlimb score = mean caliper measurement. HDL-C = HDL cholesterol. TC = total cholesterol. PON1 = paraoxonase activity. Lactonase = lactonase activity Data represent Mean ± SEM. *p < 0.05 for test of Spearman Correlation Coefficient.

Male mice with higher arthritis activity at 21 weeks had elevated serum levels of multiple cytokines compared to less arthritic females including GM-CSF, IFN-γ, IL-1β, IL-2, IL-12, and IL-17, as well as the growth factor FGF-basic, which correlated with higher arthritic scores (Fig. 2A,C). GM-CSF, IFN-γ, IL-1β, IL-12, IL-17, and FGF-basic levels correlated significantly with suppression of total and HDL cholesterol levels, low PON1 activity, and impaired HDL efflux capacity. No significant differences in serum cytokine or chemokine levels except for GM-CSF were noted between mice on an atherogenic diet compared to mice on a chow diet (Fig. 2B).

### Dysregulation in hepatic lipid metabolism genes in K/BxN mice

**Figure 3:**
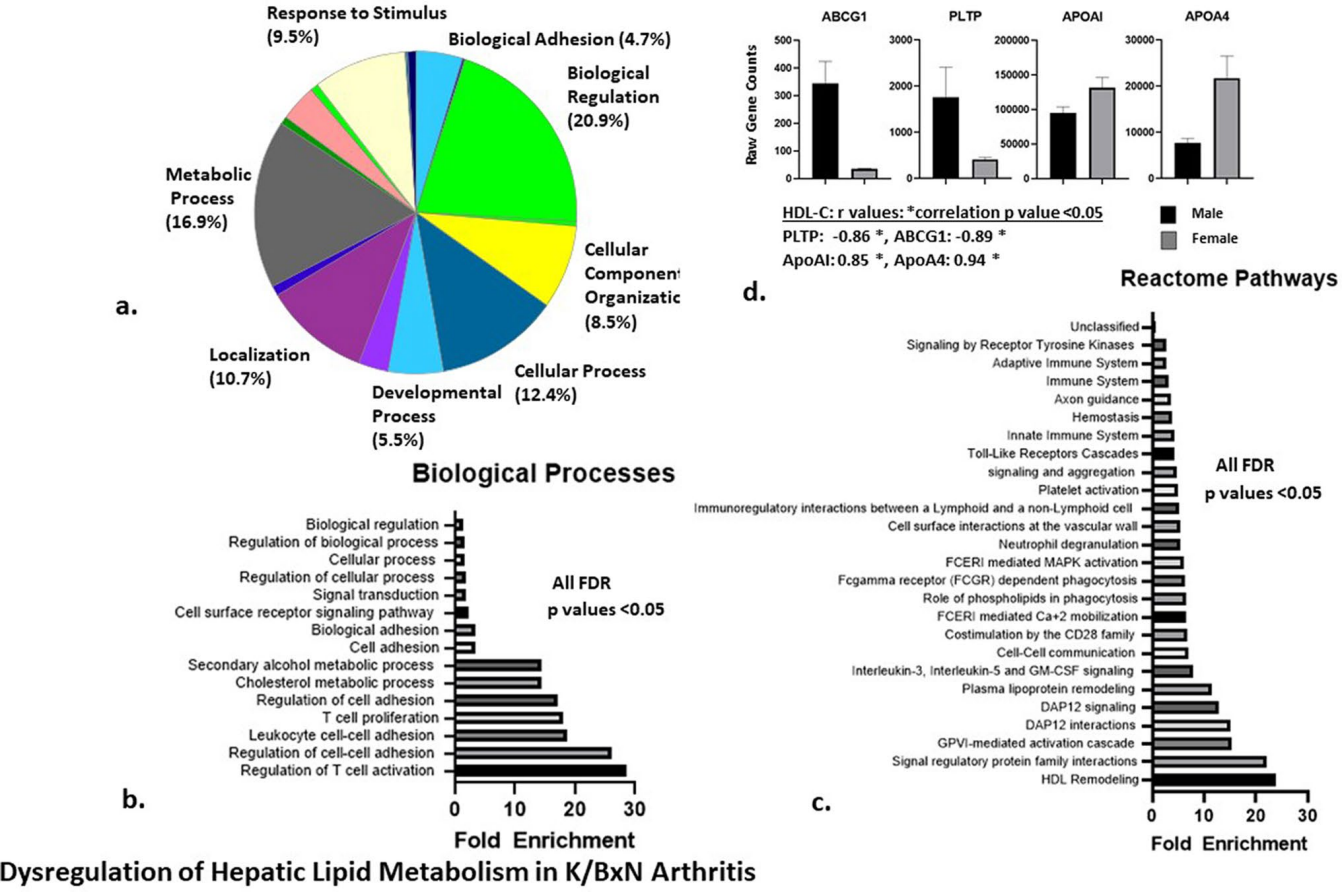
Total RNA was isolated from liver tissues and RNA sequencing analysis was performed as in described in the *Materials and Methods* section for 8 mice including 3 male and 3 female 21 week-old K/BxN mice and 2 male and 2 female WT mice controls (all on chow diet). The male K/BxN mice had higher arthritis activity compared to K/BxN female mice as shown in Fig. 1. Differences in gene expression between more arthritic male K/BxN mice and less arthritic female K/BxN mice were analyzed after controlling for non-arthritic male/female differences in WT controls. (**A**) Functional classification of the resulting gene pathways from the PANTHER GO-Slim Biological Process (BP) analysis. (**B**) PANTHER Overrepresentation Pathway BP analysis including the fold-enrichment values for the pathways. (**C**) PANTHER Reactome Process pathway overrepresentation analysis, which identified 25 gene pathways, which were significantly enriched in the gene data set. The HDL remodelling pathway was the most highly enriched pathway. (**D**) Differences in 4 individual genes related to lipid metabolism, which were identified in the HDL remodeling pathway between more arthritic males and less arthritic females after controlling for non-arthritic sex differences. Correlations between raw gene expression counts and HDL cholesterol (HDL-C) levels are shown. Statistical analyses were performed as described in the *Materials and Methods* section. Software: PANTHER version 14.0: https://www.pantherdb.org.

To evaluate mechanisms driving the dyslipidemia associated with K/BxN arthritis, RNA sequencing of liver tissue was performed in 21 week-old arthritic K/BxN mice and non-arthritic C57BL/6 controls. Differences in gene expression were examined between more arthritic K/BxN males and less arthritic K/BxN females at 21 weeks after adjusting for non-arthritic male/female gene differences in controls. Altogether, 323 genes were identified in the analysis including 277 genes upregulated and 46 genes downregulated in the more arthritic animals compared to the less arthritic animals (Supplementary Table 2). Functional biological classification of these genes revealed that 16.9% were involved in metabolic processes. This group was secondary in magnitude only to genes involved in biological regulation, which contained 20.9% of genes (Fig. 3A).

The PANTHER Reactome Process pathway overrepresentation analysis identified 25 gene pathways significantly enriched in the gene data set (FDR p values < 0.05). The HDL remodelling pathway had the highest fold-enrichment of all pathways identified (Fig. 3C). PANTHER Biological Process pathway overrepresentation analysis identified 15 enriched pathways in the gene data set (false discovery rate (FDR) p values < 0.05). The cholesterol metabolic process pathway was the sixth most enriched pathway in this analysis, following T cell activation, T-cell proliferation, and leukocyte cell adhesion pathways (Fig. 3B).

Changes in individual genes related to lipid metabolism identified in the HDL remodeling pathway included a 15-fold higher expression of ATP binding cassette transporter G1 (ABCG1) and a sevenfold higher expression of phospholipid transfer protein (PLTP) in the more arthritic males compared to less arthritic females, after adjustment for baseline sex differences (Supplementary Table 2). Out of the 323 genes identified in the expression analysis, ABCG1 was one of 18 genes most significantly different between arthritic males and less arthritic females after sex adjustment (Supplementary Table 2). Higher raw expression counts of both ABCG1 and PLTP genes correlated significantly with lower HDL-C levels (Fig. 3D). Apolipoprotein AI (apoA1) and apoA4 genes were threefold down-regulated in the more arthritic males compared to less arthritic females after adjusting for baseline male/female differences (Supplementary Table 2). Lower apoA1 and apoA4 gene expression counts correlated significantly with lower HDL-C levels (Fig. 3D). Lower apoAI expression also associated with impaired HDL anti-oxidant capacity measured by the HII (r = -0.86, p = 0.03).

### Increased bioactive lipid mediators in K/BxN mice and RA patients

**Figure 4:**
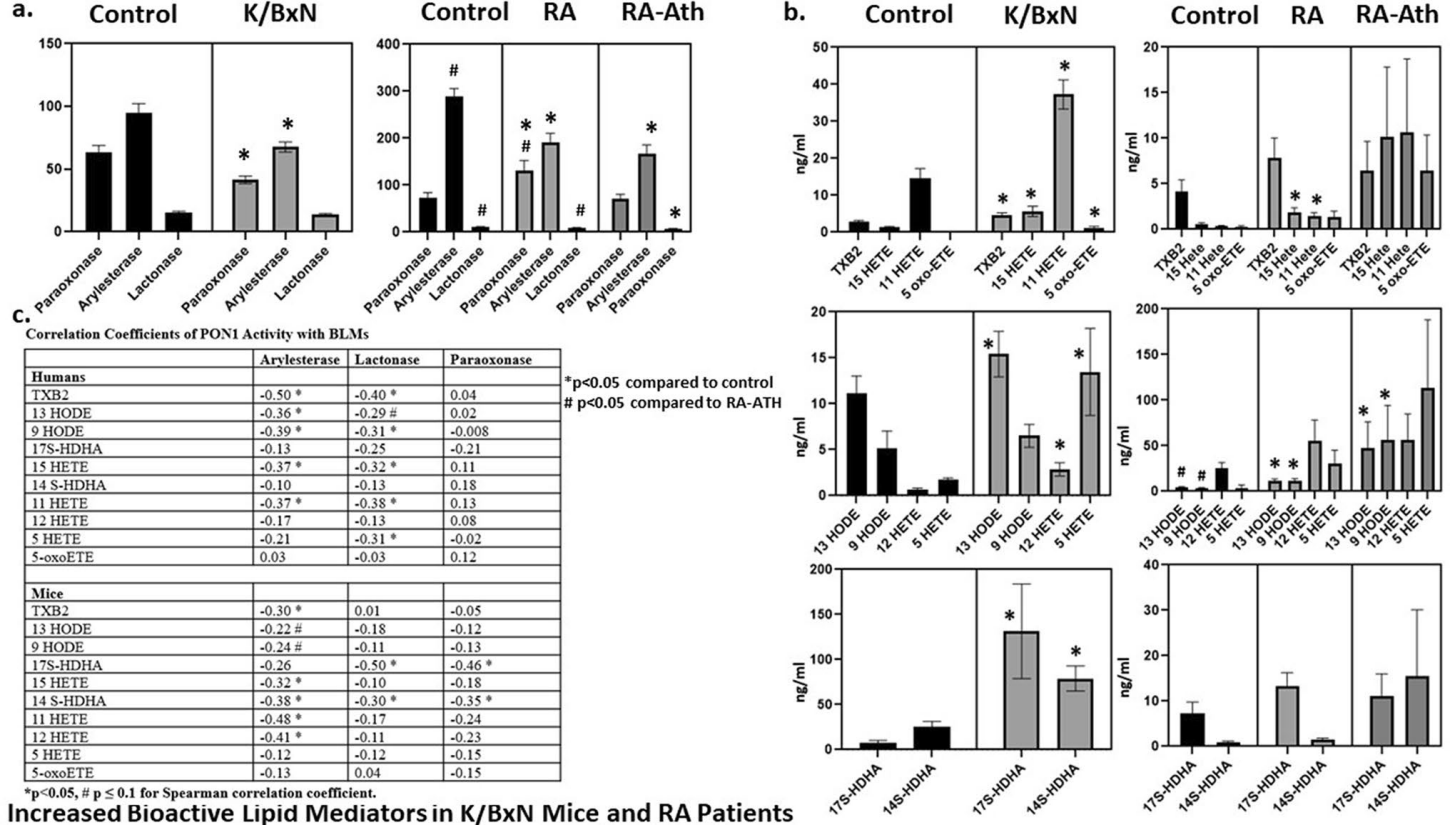
Arthritic K/BxN mice were bred from non-arthritic C57B6/Ag7 females and KRN males to evaluate bioactive lipid mediators (BLM) in murine inflammatory arthritis. All mice were maintained on a standard mouse chow diet until sacrifice at 9 weeks (n = 10; 5 M/5F), 14 weeks (n = 12; 6 M/6F), and 21 weeks (n = 7; 2 M/5F) [arthritic K/BxN mice = 29] and 14 weeks KRN (n = 10; 5 M/5F) and C57Bl/6-Ag7 (n = 10; 5 M/6F), [non-arthritic controls = 21]. 16 RA patients with atherosclerotic plaque identified on carotid ultrasound were also compared to 16 matched RA patients without plaque and 16 non-RA healthy controls. Mouse and human RA groups were compared to controls as described in the “Materials and methods”. (**A**) PON1 activities. (**B**) Bioactive lipid mediators (BLM). (C) Correlations of BLM with PON1 activities.

Oxidation products of arachidonic acid and linoleic acid including hydroxyeicosatetraenoic acids (HETES) and hydroxyoctadecadienoic acids (HODES) are important BLM involved in the immune response, which have also been directly implicated in propagation of atherosclerosis^17–19^ . A lipidomics panel of BLM including several HETES and HODES was evaluated in arthritic K/BxN mice and KRN/Ag7 non-arthritic controls. 9/10 BLM were significantly elevated in arthritic mice compared to non-arthritic controls (Fig. 4B). PON1 activity was suppressed in arthritic animals, and lower PON1 activity correlated significantly with higher levels of several BLM (Fig. 4A,C).

Healthy human controls (HC) were matched to two groups of RA patients including patients with active disease and carotid atherosclerosis (RA-ATH), and RA patients with quiescent disease and no carotid atherosclerosis (RA) (Supplementary Table 3). PON1 activity was significantly lower in both groups of RA patients compared to HC and was significantly lower in the RA-ATH group compared to the RA group by both lactonase and paraoxonase assays (Fig. 4A). Multiple BLM were higher in RA patients compared to HC, and trends were observed for highest levels in the RA-ATH group (Fig. 4B). Higher BLM correlated with lower circulating PON1 activity, similar to the mouse data (Fig. 4C).

### Overexpression of the human PON1 transgene reduces inflammatory arthritis and dyslipidemia

#### Clinical arthritis

**Figure 5:**
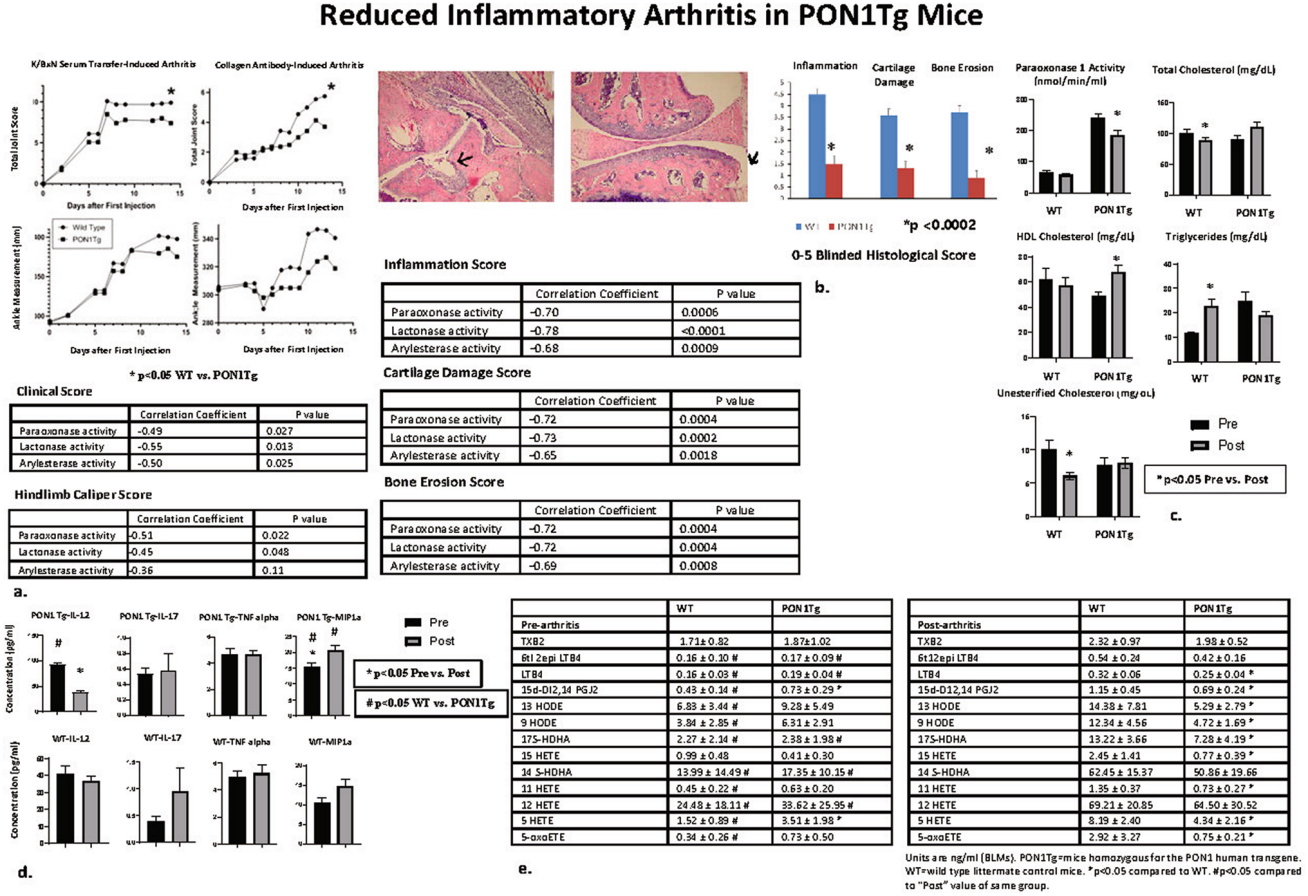
Mice homozygous for the PON1 human transgene [PON1Tg] and wild type littermate control mice [WT] were injected intraperitoneally (n = 10 per group) with either 200ul of pooled K/BxN serum on days 0 and 2 (Serum Transfer Induced Arthritis [STIA], age = 4 months, 5M/5F) or 5 mg of collagen antibody cocktail (Chondrex) on day 0, and 50ug of LPS on day 3 (collagen antibody-induced arthritis [CAIA], age = 8 months, 5M/5F). Arthritis activity was assessed using caliper measurements of hind limbs and clinical scores until sacrifice at 2 weeks as described in *Materials and Methods.* (**A**) Arthritic scores over time and negative correlations between arthritis activity measures and serum PON1 activity. (**B**) Lower histologic damage scores in PON1Tg mice compared to controls, negative correlations between histological scores and serum PON1 activity, and representative histology of one PON1Tg and one control mouse (STIA experiment). (**C**) Baseline and post- arthritis lipid profiles of PON1TG and WT mice (STIA experiment). (**D**) Serum cytokine/chemokine levels (Luminex-based 20 plex assays) with detectable analyte concentrations for the majority of mice in each group (≥ 65%; IL-12, IL-17, TNF- α, MIP-1α) (STIA experiment). (E) Pre and post-arthritis serum levels of BLM for WT and PON1Tg mice (STIA experiment).

PON1Tg mice express the human PON1 transgene in hepatic tissue and have a 2–3 fold higher serum PON1 activity compared to WT controls. K/BxN serum transfer induced arthritis (STIA) was induced in PON1Tg and WT mice per protocol^20^. Both groups developed clinical arthritis, but PON1Tg mice had significantly lower arthritis activity compared to WT mice (Fig. 5A), which was confirmed in the CAIA model of RA^21^. Higher serum PON1 activity correlated significantly with lower clinical arthritis scores (Fig. 5A).

#### Histologic arthritic disease

Joint inflammation and damage were assessed by histology of left ankle joints for all PON1Tg and WT mice induced with KBxN arthritis. PON1Tg mice had significantly lower blinded scores of joint inflammation, cartilage damage, and bone erosion compared to WT mice. Histology scores were highly correlated with serum PON1 activity as assessed by paraoxonase, lactonase, and arylesterase assays (Fig. 5B).

#### Traditional lipid profiles

WT mice had suppression of TC and HDL-C, and increases in triglycerides (TG) after arthritis induction, similar to observations in patients with active RA^15^. In contrast, PON1Tg mice had no significant changes in TC or TG but increases in HDL-C after arthritis induction (Fig. 5C). Higher HDL-C levels correlated modestly with improved HDL function measured by a lower HII (r = − 0.41, p = 0.07).

#### Cytokine/chemokine changes

Serum concentrations of cytokines and chemokines in PON1Tg and WT mice after arthritis induction for 2 weeks were too low for accurate concentration measurement for the majority of analytes assessed. Out of the cytokines/chemokines with detectable analyte concentrations for the majority of mice in each group (≥ 65%; IL-12, IL-17, TNF- α, MIP-1α) , IL-12 showed a marked difference between PON1Tg mice and WT controls. IL-12 serum levels were lower following arthritis induction in PON1Tg mice, and correlated with the observed increases in HDL-C levels in this group (r = − 0.52, p = 0.0006). No significant changes were observed in IL-12 levels following arthritis induction in WT mice. MIP-1α was increased in PON1Tg mice compared to WT both before and after arthritis induction (Fig. 5D).

#### BLM

Non-arthritic WT and PON1Tg mice had similar levels of BLM at baseline, however, WT mice had significant increases in the majority of BLM (11/13) after arthritis induction. PON1Tg mice did not have similar increases post arthritis induction, and had significantly lower levels of 9/13 BLM post-arthritis induction compared to WT mice (Fig. 5E). Lower levels of BLM correlated with lower clinical and histologic arthritis scores (r values = 0.5—0.8, p values < 0.05) for 10/13 BLM (Supplementary Table 4).

### Upregulation of the hepatic glutathione metabolic pathway in PON1Tg mice

#### Hepatic transcriptome analysis

Hepatic RNA sequencing analysis of K/BxN mice with chronic arthritis of 21 weeks duration revealed marked abnormalities in lipid metabolism gene expression, which correlated with arthritis severity. In the current analysis, we did not find similarly marked hepatic lipid pathway changes in younger mice with arthritis induction for only 2 weeks. However, in total 151 genes upregulated and 87 genes downregulated, were differentially expressed between PON1Tg mice compared to WT mice after adjustment for non-arthritic PON1Tg/WT gene differences. (Supplementary Table 5).

Unexpectedly, the glutathione pathway was consistently upregulated in PON1Tg mice compared to controls following arthritis induction in multiple analyses, after adjustment for non-arthritic baseline differences. In KEGG pathway analysis, the hepatic glutathione metabolism pathway was 14-fold upregulated in PON1Tg compared to WT mice after arthritis induction, controlling for baseline expression differences (Bonferroni p value < 1.5E−04) (Table 1). Similarly, in the Gene Ontology (GO) Molecular Function Pathway analysis, the glutathione transferase activity pathway was 20-fold upregulated in PON1Tg mice compared to WT mice following arthritis induction (Table 1). Increased expression of 7/8 genes in the glutathione pathway was significantly associated with lower circulating BLM, which correlated with less arthritic disease (r values = − 0.5–0.8, p values < 0.05; correlations of raw gene expression counts/various BLM) (Supplementary Table 6).

**Table 1:** Hepatic Transcriptome Analysis of Arthritic PON1Tg Mice Compared to Arthritic Control Mice after Adjustment for Baseline Non-arthritic PON1Tg/Control Differences.

ID	Genes	Fold Enrichment	P value	Adjusted P value
**Kyoto Encyclopedia of Genes and Genomes (KEGG ) Pathway**				
mmu00480:Glutathione metabolism	GSTM1, MGST3, GSTA2, GSTM2, GSTM3, GPX6, GSTT3, GSTM6	14.21	1.12E−06	1.47E−04
mmu00980:Metabolism of xenobiotics by cytochrome P450	GSTM1, MGST3, GSTA2, GSTM2, GSTM3, GSTT3, GSTM6	10.69	4.12E−05	0.0027
mmu00982:Drug metabolism—cytochrome P450	GSTM1, MGST3, GSTA2, GSTM2, GSTM3, GSTT3, GSTM6	10.36	4.92E−05	0.0021
mmu05204:Chemical carcinogenesis	GSTM1, MGST3, GSTA2, GSTM2, GSTM3, GSTT3, GSTM6	7.44	3.13E−04	0.0102
mmu03320:PPAR signaling pathway	CYP4A10, CYP4A31, CYP4A14, CPT1A, PLTP	6.11	0.0085	0.2012
mmu00830:Retinol metabolism	CYP4A10, DHRS3, CYP4A31, CYP4A14, RETSAT	5.49	0.0123	0.2371
mmu00071:Fatty acid degradation	CYP4A10, CYP4A31, CYP4A14, CPT1A	7.98	0.0131	0.2187
mmu04976:Bile secretion	NCEH1, AQP8, NR0B2, SLC10A2	5.51	0.0347	0.4394
mmu00590:Arachidonic acid metabolism	CYP4A10, GPX6, CYP4A31, CYP4A14	4.39	0.0608	0.5984
**Gene Ontology (GO) Molecular Function Pathway**				
GO:0004364 ~ glutathione transferase activity	GSTM1, MGST3, GSTA2, GSTM2, GSTM3, GSTT3, GSTM6	20.21	1.03932E−06	0.0006
GO:0016765 ~ transferase activity, transferring alkyl or aryl (other than methyl) groups	GSTM1, MGST3, GSTA2, GSTM2, GSTM3, GSTT3, GSTM6	10.59	4.92144E−05	0.0149
GO:0018685 ~ alkane 1-monooxygenase activity	CYP4A10, CYP4A31, CYP4A14	47.64	0.0016	0.2746
GO:0016713 ~ oxidoreductase activity, acting on paired donors, with incorporation or reduction of molecular oxygen, reduced iron-sulfur protein as one donor, and incorporation of one atom of oxygen	CYP4A10, CYP4A31, CYP4A14	28.59	0.0046	0.5053
GO:0042803 ~ protein homodimerization activity	CHKA, PTPRE, MVD, HMGCS1, BC029214, NR0B2, MID1, PEX11A,	2.03	0.0094	0.6852
	APOA4, GSTM1, GSTM2, GSTM3, SYNE1, PYCARD, TDG, GAMT, TERT			
GO:0015293 ~ symporter activity	SLC15A2, SLC17A4, SLC13A2, SLC22A5, SLC36A4, SLC10A2	3.97	0.0176	0.8350
GO:0004416 ~ hydroxyacylglutathione hydrolase activity	PNKD, HAGHL	63.53	0.0310	0.9353
GO:0042895 ~ antibiotic transporter activity	SLC15A2, SLC22A5	63.53	0.0310	0.9353
GO:0046983 ~ protein dimerization activity	CHKA, PTPRE, MVD, TAF4B, MLXIPL, HMGCS1, NR0B2, BC029214, MID1, PEX11A,	1.63	0.0310	0.9091
	APOA4, GSTM1, GSTM2, NPAS2, GSTM3, SYNE1, PYCARD, TDG, SOX18, GAMT, TERT			
GO:0008324 ~ cation transmembrane transporter activity	KCNT2, SLC17A4, NDUFA4L2, KCNA2, SLC41A3, MCU, SLC13A2,	1.99	0.0386	0.9301
	SLC22A5, SLC10A2, CHRNA2, KCNIP3, PKD2L2			
**Gene ontology (GO) Biological Process Pathway**				
GO:0006749 ~ glutathione metabolic process	GSTM1, GSTA2, GSTM2, ETHE1, GSTT3, HMGN5	10.21	0.0003	0.6030
GO:0035634 ~ response to stilbenoid	APOA4, GSTA2, SAA2, FGL1	14.66	0.0024	0.9791
GO:0006575 ~ cellular modified amino acid metabolic process	GSTM1, GSTA2, GSTM2, ETHE1, CRTAP, GSTT3, HMGN5	4.98	0.0028	0.9506
GO:0006720 ~ isoprenoid metabolic process	APOA4, DHRS3, MVD, HMGCS1, PDE3A, RETSAT	6.08	0.0030	0.9108
GO:0015850 ~ organic hydroxy compound transport	APOA4, GCK, AQP8, KCNA2, LIPG, ABCA2, SLC10A2, PLTP	3.99	0.0040	0.9198
GO:0006629 ~ lipid metabolic process	CHKA, PLP1, NCEH1, MVD, HMGCS1, MLXIPL, ABCA2, PDE3A, ST8SIA3, GPCPD1, PROX1, CPT1A,	1.88	0.0042	0.8889
	CD74, APOA4, CYP4A10, ST6GALNAC3, DHRS3, SAA1, FITM2, ELOVL2, LIPG, FGL1, PLTP, RETSAT			
GO:0006066 ~ alcohol metabolic process	APOA4, CHKA, DHRS3, MVD, SAA1, HMGCS1, ABCA2, FGL1, RETSAT	3.42	0.0050	0.8942
GO:1901615 ~ organic hydroxy compound metabolic process	APOA4, CHKA, DHRS3, MVD, SAA1, PNKD, HMGCS1, ABCA2, FGL1, PLTP, RETSAT	2.79	0.0062	0.9131
GO:0008203 ~ cholesterol metabolic process	APOA4, MVD, SAA1, HMGCS1, ABCA2, FGL1	5.10	0.0064	0.8961
GO:0032374 ~ regulation of cholesterol transport	APOA4, LIPG, ABCA2, PLTP	10.03	0.0072	0.8993

In examination of PON1Tg versus WT mice prior to arthritis induction, the glutathione pathway was identified in pathway analyses (Supplementary Table 7), although it was not similarly upregulated as in the PON1Tg arthritic mice and was not statistically significant. QPCR analysis was performed in a second group of PON1Tg mice and controls. This analysis also did not demonstrate significant expression differences in glutathione pathway genes between non-arthritic PON1Tg and WT groups (data not shown). However, following arthritis induction, the hepatic glutathione pathway genes glutathione peroxidase 6 (GPX6), glutathione S-transferase Mu 2 (GSTM2), and GSTM6 were upregulated in PON1Tg but not in WT mice compared to non-arthritic controls (Supplementary Fig. 1), These data were similar to our initial RNA sequencing analyses. No significant differences between groups were noted in expression of GSTM1, GSTM3, or GSTA2.

#### Association of the PON1 and glutathione pathways in the hybrid mouse diversity panel (HMDP)

In order to further evaluate a relationship between PON1 and the glutathione pathway, we examined hepatic gene transcription data from the hybrid mouse diversity panel (HMDP). HMDP is a collection of approximately 100 well-characterized inbred strains of mice exhibiting substantial diversity of most cardiovascular and metabolic traits relevant to human disease^22^. We examined specific glutathione pathway genes including GSTM6, GSTM3, and GPX6, which were differentially expressed in PON1Tg mice compared to WT mice after arthritis induction in our initial pathway analysis. We reviewed the top 1000 genes, which correlated with transcript levels of GSTM6, GSTM3, and GPX6 in HMDP livers. The PON1 gene was identified as one of the top 1000 genes (#540/1000) correlating with GSTM6 transcript levels (r = 0.376).

We also examined the top 1000 genes correlated with PON1 transcript levels in HMDP livers and found that multiple glutathione pathway genes correlated with PON1 transcript expression including Gsto1, Gstm6, Gsta3, and Gstt1 (Supplementary Table 8). In addition, PON1 transcript levels in HMDP livers correlated with other differentially expressed genes in our initial pathway analysis including DHRS3, AQP8, and NR0B2 (r values = 0.337, 0.389, and 0.388 respectively).

#### Increased hepatic PON2 and PON3 protein expression in PON KO mice

We hypothesized that knock out of the PON1 gene would result in more severe arthritic disease following arthritis induction. However, no difference in arthritis activity was noted between PON1KO mice and WT mice following arthritis induction with K/BxN serum transfer or collagen antibodies. No differences in cholesterol levels were noted between the groups after arthritis induction, however, cholesterol levels and PON1 activity decreased following arthritis induction, consistent with our earlier findings in K/BxN mice (Supplementary Fig. 2).

PON1KO mice have previously been shown to be paradoxically protected from pseudomonal infection due to hepatic upregulation of PON2 and PON3 proteins^23^. Therefore, we performed western blot analysis of liver tissue for both PON2 and PON 3 proteins in PON1 KO and WT mice. Levels of PON2 and PON3 proteins were significantly higher in livers from PON1KO mice compared to WT controls following arthritis induction (Supplementary Fig. 3). Further work evaluating roles of other PON proteins in inflammatory arthritis may be warranted.

## Discussion

The lipid “paradox” was first described in epidemiologic studies of patients with active RA who had high atherosclerotic risk despite low circulating total cholesterol levels^24^. Mechanisms for the suppression of cholesterol in the setting of active RA remain unclear. However, impairment in HDL function and low PON1 activity have been implicated as alternative mechanisms by which active RA may increase CV risk, irrespective of circulating total cholesterol levels^6,13^.

In the current work, arthritic disease activity was closely associated with dyslipidemia in K/BxN mice with chronic arthritis. PON1 activity was markedly decreased, and multiple BLM were elevated in association with arthritis activity. Cholesterol levels were also suppressed, and HDL was dysfunctional, similar to the dyslipidemia noted in active RA patients. Low circulating HDL cholesterol levels correlated significantly with impaired HDL function in several experiments (Fig. 1A,C, PON1Tg experiments).

Surprisingly, an atherogenic diet did not influence cholesterol levels or PON1 activity in arthritic mice (Fig. 1B) in contrast to the well-studied increases in cholesterol and suppression of PON1 activity with an atherogenic diet in non-arthritic mice^25^. These data highlight the profound effect of arthritic inflammation relative to diet on lipid metabolism in arthritic K/BxN mice, and further emphasizes the need for better understanding of the altered lipid metabolism in RA.

Hepatic RNA sequencing analysis confirmed alterations of lipid metabolism pathways, which associated with arthritis activity in K/BxN mice. The HDL remodeling pathway was the most highly enriched pathway of any reactome pathway associated with arthritis activity, consistent with the finding of impaired function of HDL in arthritic animals. These data parallel work in humans that demonstrated the presence of dysfunctional HDL and altered HDL proteome in patients with active RA^26^.

Biological process pathway analysis also identified the cholesterol metabolic pathway as one of the most highly enriched hepatic pathways in arthritic mice. Upregulation of hepatic ABCG1 and PLTP genes was associated with more severe arthritic disease in K/BxN mice, and higher gene expression counts correlated with lower serum HDL-C levels. These findings are consistent with previous data, which reported low circulating HDL-C levels associated with hepatic overexpression of ABCG1 or PLTP genes by adenovirus in non-arthritic mice^27,28^. These data may suggest potential mechanisms for the so-called “lipid paradox” in RA patients by alterations in hepatic lipid metabolism associated with arthritis activity.

Lipid peroxidation reactions associated with inflammatory conditions such as RA produce BLM, which have important roles in the propagation of both atherosclerosis and rheumatoid arthritis. Oxidation products of arachidonic and linoleic acid, HETES and HODES, are BLM linked to angiographic evidence of coronary artery disease in humans^17^. We previously reported elevated levels of HETES and HODES in dysfunctional HDL and synovial fluid from active RA patients^5^. In the current work, several BLM including HETES and HODES were elevated in circulation of arthritic mice and humans compared to controls and correlated with higher arthritis disease activity measures. RA patients with atherosclerosis had the highest levels of 8/10 BLM studied.

PON1 is an HDL-associated protein which inhibits lipid peroxidation and has been associated with lower atherosclerotic risk in population studies and reduced atherosclerosis in animal models^12,14^. Work by Teiber et al. recently suggested that PON1 may directly hydrolyze certain BLM including biologically active δ lactone eicosanoids^11^. The current work is the first study to investigate the role of PON1 in a mouse model of inflammatory arthritis. In our study, higher circulating PON1 activity correlated with lower BLM in both arthritic mice and humans. Overexpression of the human PON1 transgene prevented increases in multiple BLM following arthritis induction and reduced both clinical and histologic arthritic disease. In particular, PON1Tg mice had lower post-arthritis levels of 5 HETE and 15HETE, 2 BLM linked previously to both RA and CV risk^18,19,29^.

Hepatic RNA sequencing analysis of arthritic PON1Tg mice compared to controls identified an unexpected, but consistent upregulation in the hepatic glutathione pathways in PON1Tg mice, which correlated with lower BLM following arthritis induction. We did not find dysregulation in the large number of hepatic lipid pathways, which were identified in K/BxN mice with chronic arthritis for over 4 months. This difference may relate to the much shorter duration of arthritis activity of two weeks in the PON1Tg experiment. However, the relationship noted between the PON1 and glutathione pathways was consistent in both KEGG and molecular function pathway analysis, and further validated in the HMDP, which identified correlations between both hepatic gene and transcript levels of several PON1 and glutathione pathway proteins.

The glutathione pathway is well recognized as a global “anti-oxidant” system, and previous work in rat adjuvant-induced arthritis suggests that lipid peroxides formed in arthritic joints are released into circulation, trapped by the liver, and metabolized by the glutathione pathway^30^. However, as inflammatory arthritis continues, the glutathione pathway is depleted, including decreases in glutathione transferase enzyme activity, which allows propagation of arthritic disease. In the current studies, PON1Tg mice had upregulation of the hepatic glutathione pathway, lower BLM, and reduced arthritic disease activity and damage.

To our knowledge, the current work is the first study to link PON1 to upregulation of the hepatic glutathione system and improvement in inflammatory arthritis. In humans, a deletion polymorphism in the glutathione s-transferase gene has been associated with greater risk of RA, as well as with more severe, anticitrullinated protein antibody (ACPA) positive RA^31^. Interestingly, Garcia-Heredia et al. previously reported dysregulation in the hepatic glutathione pathway in PON1 deficient mice with fatty liver disease^32^.

Yamashita and colleagues first reported immunomodulatory effects of PON1 in a mouse model of inflammatory colitis via inhibition of IFN-γ production from CD4 T cells^33^. The K/BxN model of RA used in this work is a model of the “effector” phase of RA-like disease and does not require lymphocytes^20^. In the current study, serum cytokine and chemokine levels including IFN-γ were generally low in both mouse groups while marked differences in BLM were noted. Of the cytokines/chemokines with measurable concentrations, IL-12 levels showed significant decreases following arthritis induction in PON1Tg mice, which correlated with the observed HDL-C increases in these animals, which were not seen in WT mice. This relationship was also noted in mice with chronic K/BxN arthritis in which IL-12 was the cytokine most closely correlated with HDL-C levels. IL-12 has previously been described as both a pro-inflammatory and anti-inflammatory cytokine dependent on the stage of arthritic disease in mouse models^34,35^. Further investigation of its role in lipid metabolism in the setting of RA may be warranted.

PON1 KO mice did not have worsened arthritis following induction with K/BxN serum or collagen antibody transfer. The arthritis intensity was high in both PON KO and WT mice, and cholesterol levels were suppressed after arthritis induction, which is consistent with marked arthritis activity. It is possible that the intensity of inflammation prevented sufficient “potential” to observe further worsening of arthritis with KO of the PON1 gene. However, in addition, the PON gene family includes PON2 and PON3 proteins in addition to PON1, which also have anti-oxidant functions linked to protection against oxidative stress and atherosclerosis^36^. Hepatic expression of both PON2 and PON3 proteins were increased in PON1 KO compared to WT mice. While further investigation is warranted, we hypothesize that the anti-oxidant effects of these PON proteins may have prevented worsened arthritis in KO mice. In previous work by Ozer et al., PON1 KO mice had a paradoxical, slightly improved survival following intraperitoneal challenge with P aeruginosa, which was also attributed to increases in PON2 and PON3 protein expression^23^.

In summary, the current work is the first study to evaluate the role of the HDL-associated protein, PON1 in a mouse model of RA. The work describes a novel, protective role of PON1 in inflammatory arthritis through upregulation of the glutathione system and reduction in BLM. Insights into the so-called “lipid paradox” were also gained, suggesting that alterations in hepatic lipid metabolism may contribute to suppression of cholesterol levels and impairment in HDL function noted in RA patients.

Atherosclerotic vascular disease remains a leading cause of morbidity and mortality in RA patients despite current immunosuppressive therapies. Additional agents targeting novel pathways in inflammatory arthritis are needed, and PON1 may warrant further investigation for its ability to reduce vascular risk as well as arthritic disease in patients with RA.

## Materials and methods

### Animals

C57BL/6 mice expressing the transgenic T cell receptor KRN and C57BL/6 mice expressing the MHC class II molecule A^g7^ (A^g7^ C57BL/6) were a gift of Drs. Christophe Benoist and Diane Mathis. Non-obese diabetic (NOD) female mice expressing the MHC class II molecule A^g7^ were purchased from Jackson lab. K/BxN mice were generated by crossing male KRN mice with NOD female mice or A^g7^ C57BL/6 mice. Mice overexpressing the human PON1 transgene (C57BL/6 background), [PON1 transgenic (PON1Tg) mice], PON1 knockout (KO) mice, and wild type controls were as previously described^14,37^. Standard mouse chow diet and atherogenic diet (15.8% fat, 1.25% cholesterol) were from Teklad, Harlan (catalog no T7013M15 and TD.94059, respectively). K/BxN serum transfer arthritis (STIA) and collagen antibody-induced arthritis (CAIA) protocols were performed as described^20,21^ and further details of the experiments are provided in figure legends. For blood collection, mice were fasted overnight, and blood was collected by orbital puncture. The animal protocols were approved by the Animal Research Committee at UCLA and the methods were carried out in accordance with the approved guidelines.

### Humans

RA patients and healthy controls (HC) were recruited from the rheumatology offices at the University of California, Los Angeles (UCLA). All subjects gave written informed consent for the study under a protocol approved by the Human Research Subject Protection Committee at UCLA. All methods were carried out in accordance with relevant guidelines and regulations for human subjects. 16 RA patients with carotid atherosclerosis (RA-ATH), 16 RA patients without carotid ATH (RA), and 16 HC were included. All RA patients met the American College of Rheumatology criteria for RA, which was verified by chart review. Blood was collected in heparinized tubes (Becton Dickinson) and stored at − 80 °C. Assessment of inflammatory markers including high-sensitivity C-reactive protein (HSCRP) and Westergren erythrocyte sedimentation rate (ESR) and fasting lipid profiles were measured in the UCLA clinical laboratory using standard methods.

### Carotid ultrasound imaging

A standard protocol including B (brightness)-mode grey scale, color and spectral Doppler techniques was used to study the carotid arteries of all RA patients as previously reported^13^.

### Clinical arthritic assessments

Arthritis was assessed by a blinded assessor using calipers (Long Island Indicator Service) to measure the ankle width of the hindlimbs, and using a clinical disease severity score with a maximum score of 12 for 4 paws as follows: score 0 = no swelling, 1 = swollen wrist/ankle, 2 = swelling extending to forepaw/hindpaw, 3 = swelling extending to digits or accompanied by joint rigidity.

### Histologic assessments

Cross-sections of the left ankle joints were stained with H&E and scored by an independent and blinded observer for inflammatory activity, cartilage damage, and bone erosion on a 0–5 point scale using an established semiquantitative scoring system as described previously^38^.

### PON1 activity

Paraoxonase activity was quantified using paraoxon as the substrate and measuring the increase in the absorbance at 405 nm due to the formation of 4-nitrophenol over a period of 12 min (at 20 s intervals) as previously described^13^. A unit of PON1 activity was defined as the formation of 1 nmol of 4-ntirophenol per minute per milliliter of sample used. Arylesterase activity was quantified using phenylacetate as the substrate and measuring the increase in the absorbance at 270 nm spectrophotometry over a 2 min period at 15 s intervals as described previously^39^. One unit of arylesterase activity was defined as 1 micromole of phenylacetate hydrolyzed per minute per milliliter of sample used. Lactonase activity was quantified using dihydrocumarin as the substrate. Dihydrocumarin hydrolysis rate was measured at 270 nm every 15 s for 10 min as described previously^39^. One unit of lactonase activity was defined as 1 micromole of dihydrocumarin hydrolyzed per minute per milliliter of sample used.

### Cholesterol efflux by HDL

The cholesterol efflux function of isolated HDL was measured by its ability to efflux ^3^H-cholesterol from mouse macrophage RAW264.7 cells as previously published^40^.

### Inhibition of lipid oxidation by HDL

The anti-oxidant function of HDL was measured by its ability to inhibit oxidation of LDL, using a previously published cell free assay, which measures the change in fluorescence intensity as a result of oxidation of 2′,7′-dichlorodihydrofluorescein diacetate (H2DCFDA) (ThermoFisher Scientific) to 2′,7′-dichlorofluorescein (DCF) in incubations with a standard LDL in the absence or presence of the test HDL^41,42^.

### Chemokine/cytokine analyses

Serum cytokine and chemokine levels were assessed using Luminex-based 20 plex assays as per standard protocols according to the Manufacturer’s instructions (Fisher Scientific).

### Serum lipid profiles

Standard serum lipid profiles were performed as per previously published protocols^43,44^.

### Bioactive lipid mediator analysis

Mass spectrographic analysis was performed on a SCIEX 5500 QTrap run in negative ion mode as described previously and controlled by Analyst 1.6.2 software^45^.

### Hepatic RNA extraction

Mice were administered an overdose of isoflurane anesthesia and organs harvested as described previously ^46^. Total RNA from liver tissues was isolated with the Quiagen RNeasy Plus Mini Kit (Qiagen, Hilden, Germany).

### RNA sequencing

Libraries for RNA-Seq were prepared with KAPA Stranded mRNA-Seq Kit. The workflow consists of mRNA enrichment, cDNA generation, and end repair to generate blunt ends, A-tailing, adaptor ligation and PCR amplification. Different adaptors were used for multiplexing samples in one lane. Sequencing was performed on Illumina HiSeq 3000 for 1 × 50 run. Data quality check was done on Illumina SAV. Demultiplexing was performed with Illumina Bcl2fastq2 v 2.17 program.

### Quantitative real-time PCR

Quantitative real-time PCR (qPCR) was performed using the CFX 96 Touch Real Time PCR Detection System (Bio-Rad laboratories, Inc., Hercules, CA). For cDNA synthesis, 1 µg of total RNA was transcribed using iScript cDNA synthesis Kit (Bio-Rad laboratories, Inc., Hercules, CA) and qPCR was performed using iQ SYBR Green Supermix (Bio-Rad laboratories, Inc., Hercules, CA) following the manufacturer’s protocols. Primer pair sequences are listed in Supplementary Table 1. The PCR was conducted using the following parameters: 95 °C for 5 min, and 35 cycles at 95 °C for 15 s, and 60 °C for 10 s and 72 °C for 1 min. Acquired data were analyzed by CFX Manager 3.1 Software (Bio-Rad laboratories, Inc., Hercules, CA). All PCR assays were performed in duplicate and the genes of interest were normalized to the housekeeping gene, GAPDH, from the same sample.

### Western blot

Protein extracts (50 mg) isolated from 6 WT and 6 PON1KO mouse livers were resolved by 4–15% SDS-PAGE, transferred onto nitrocellulose membranes and blocked in TBS containing 3% milk protein for 1 h. Mouse PON2 and PON3 antibodies were used at 1:500 dilution (R&D Systems).

### Statistical analysis

Data were analysed using JMP Pro 13.0 (SAS Institute Inc., Cary, NC, USA). Groups were compared using Student’s *t* test for continuous variables and the chi-square test of association for categorical variables, along with Fisher’s exact test for small sample sizes. When needed, nonparametric Wilcoxon rank-sum tests were used to analyse continuous variables. Correlations between variables were evaluated using the Pearson’s correlation coefficient for normally distributed data and Spearman’s correlation coefficient for nonparametric data. The significance level was pre-specified at p < 0.05.

Differential gene expression analysis was performed using Bioconductor package LIMMA (linear models for microarray data)^47,48^ for normalized log2-transformed counts of RNA-Seq data. LIMMA was used in conjunction with voom, which weighs the mean–variance relationship of the log-counts, needed for accurate generalized linear modeling. A candidate list of differential expressed genes were identified based on an absolute fold change > 3.0 [analysis 1-Fig. 3, Supplementary table 2] or > 2.0 [analysis 2- Table 1, Supplementary table 5]. Benjamini–Hochberg adjusted p-values of LIMMA’s moderated t-test [adjusted p < 0.05; analysis 2-Table 1, Supplementary table 5 or adjusted p < 0.10; analysis 1-Fig. 3, Supplementary table 2], were used as cut-off values due to the exploratory nature of the analysis.

PANTHER annotation version 14.0 was used for pathway analysis. PANTHER analysis type overrepresentation tests (Released 20181113) were performed for reactome pathways and GO-Slim Biological Process pathways. KEGG pathway analysis was performed using the previously developed KEGG database^49^.

## Supplementary information

Supplementary legends

Supplementary information
