# Supplementary material for: Suppression of inflammatory arthritis in human serum paraoxonase 1 transgenic mice
Source: Sci Rep. 2020 Oct 8;10:16848. doi: 10.1038/s41598-020-74016-w (PMC7546628; doi:10.1038/s41598-020-74016-w)
Supplement: Supplementary file 1 — Supplementary legends [file 41598_2020_74016_MOESM1_ESM.docx]

**Supplementary Figure 1.** Total RNA from liver tissues was isolated from non-arthritic PON1Tg mice (n=4, 15-16 weeks, 2M/2F) and wild type control mice WT (n=4, 15-16 weeks, 2M/2F) and arthritic PON1Tg mice (n=4, 15-16 weeks, 1M/3F) and WT mice (n=4, 15-16 weeks, 1M/3F), which had been injected intraperitoneally 2 weeks prior to sacrifice with 200ul of pooled K/BxN serum on days 0 and 2. Quantitative real-time PCR (qPCR) was performed as described in the *Materials and Methods* section. All gene expression data was normalized to the respective non-arthritic WT or PON1Tg control group. Means ± standard error of the mean (SEM) are shown.

**Supplementary Figure 2.**

(A). PON1 knockout mice [PON1KO] and wild type littermate control mice [WT] were injected intraperitoneally with either 200ul of pooled K/BxN serum on days 0 and 2 (Serum Transfer Induced Arthritis [STIA]) (n = 10 per group, 5M/5F, 3 month old) or 5mg of collagen antibody cocktail (Chondrex) on day 0, and 50ug of LPS on day 3 (collagen antibody-induced arthritis [CAIA]), (n=11 KO, 4M/7F and n= 9 WT, 3M/6F, 8 months old). Arthritis activity was assessed using caliper measurements of hind limbs and clinical scores until sacrifice at 2 weeks. (B) Laboratory assessments in CAIA mice. (C) Laboratory assessments in STIA mice. * p <0.05 compared to baseline values.

**Supplementary Figure 3.**

[PON1KO] and wild type littermate control mice [WT] (n= 6 per group, 3 month-old) were injected intraperitoneally with 200ul of pooled K/BxN serum on days 0 and 2 (Serum Transfer Induced Arthritis [STIA]) and sacrificed after 2 weeks. Total proteins were extracted from liver and PON2 and PON3 were detected using western blotting as described in the method sections. Representative western blot analysis (A), Quantification data using image J software; means ± standard error of the mean (SEM) (B), Original whole western blot shown for PON3 (C), Vinculin (D), PON2 (E), and Vinculin (F).
