# Supplementary material for: Suppression of inflammatory arthritis in human serum paraoxonase 1 transgenic mice
Source: Sci Rep. 2020 Oct 8;10:16848. doi: 10.1038/s41598-020-74016-w (PMC7546628; doi:10.1038/s41598-020-74016-w)
Supplement: Supplementary file 2 — Supplementary information [file 41598_2020_74016_MOESM2_ESM.pdf]

Suppression of Inflammatory Arthritis in Human Serum Paraoxonase 1 Transgenic Mice

Christina Charles-Schoeman*[1], Jennifer Wang[1], Ani Shahbazian[1], Yuen Yin Lee[1], Xiaoyan Wang[2], Victor Grijalva[3], Ernest Brahn[1], Diana M. Shih[3], Asokan Devarajan[3], Christy Montano[1], Aldons J. Lusis[3,4], and Srinivasa T. Reddy[3]

From the Division of Rheumatology, Department of Medicine[1], the Department of Medicine Statistics Core[2], the Division of Cardiology Department of Medicine[3], Departments of Human Genetics, Microbiology, Immunology, and Molecular Genetics[4], at the University of California, Los Angeles, Los Angeles, CA 90095.

SUPPLEMENTARY DATA SECTION

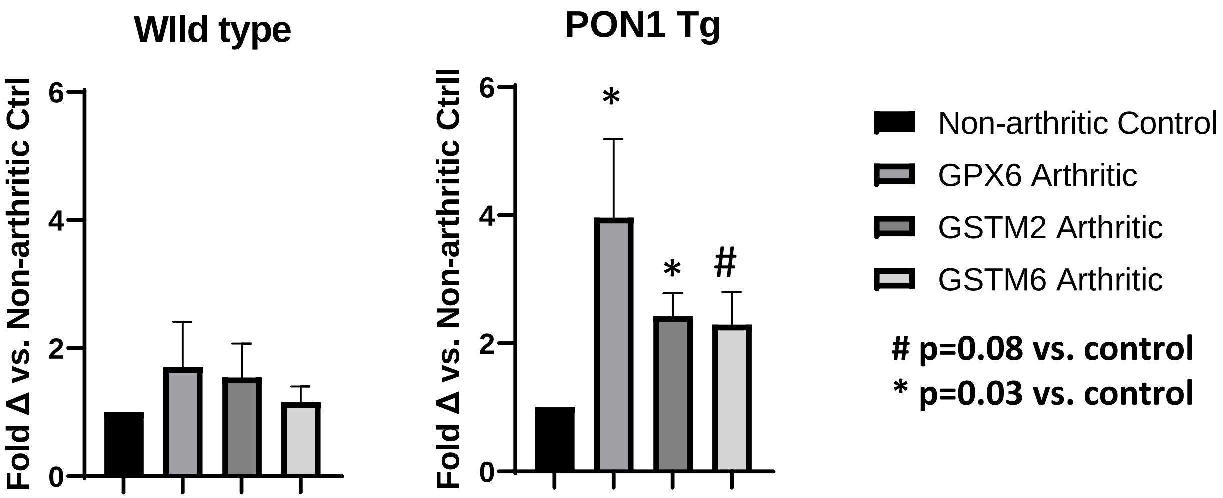

Supplementary Figure 1. Increased Hepatic Glutathione Gene Expression in Arthritic PON1Tg Mice

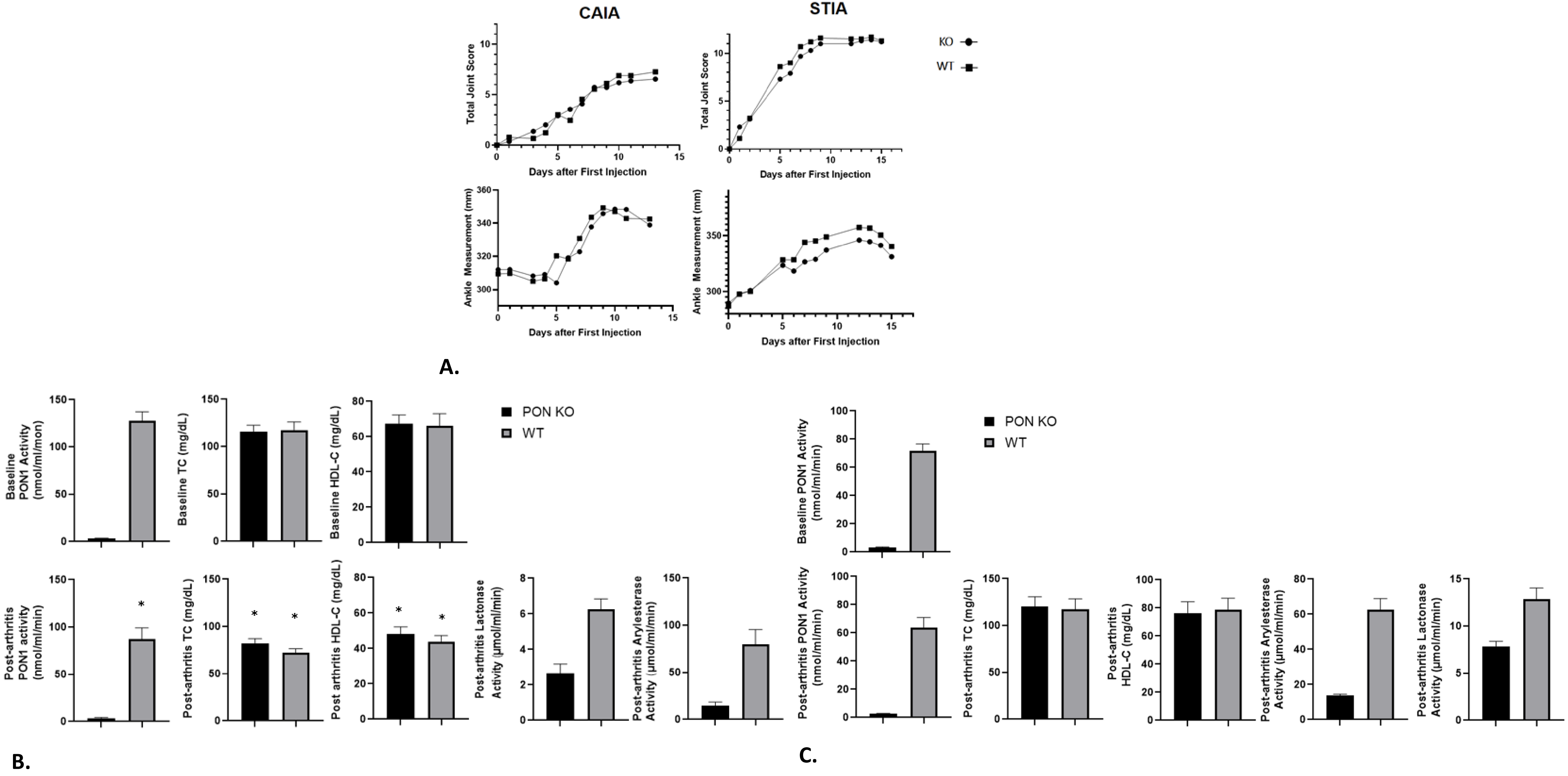

Supplementary Figure 2. PON1 Knockout Mice and Controls Following Arthritis Induction.

Supplementary Figure 3. Increased Hepatic PON2 and PON3 Protein Expression in Arthritic PON1KO Mice

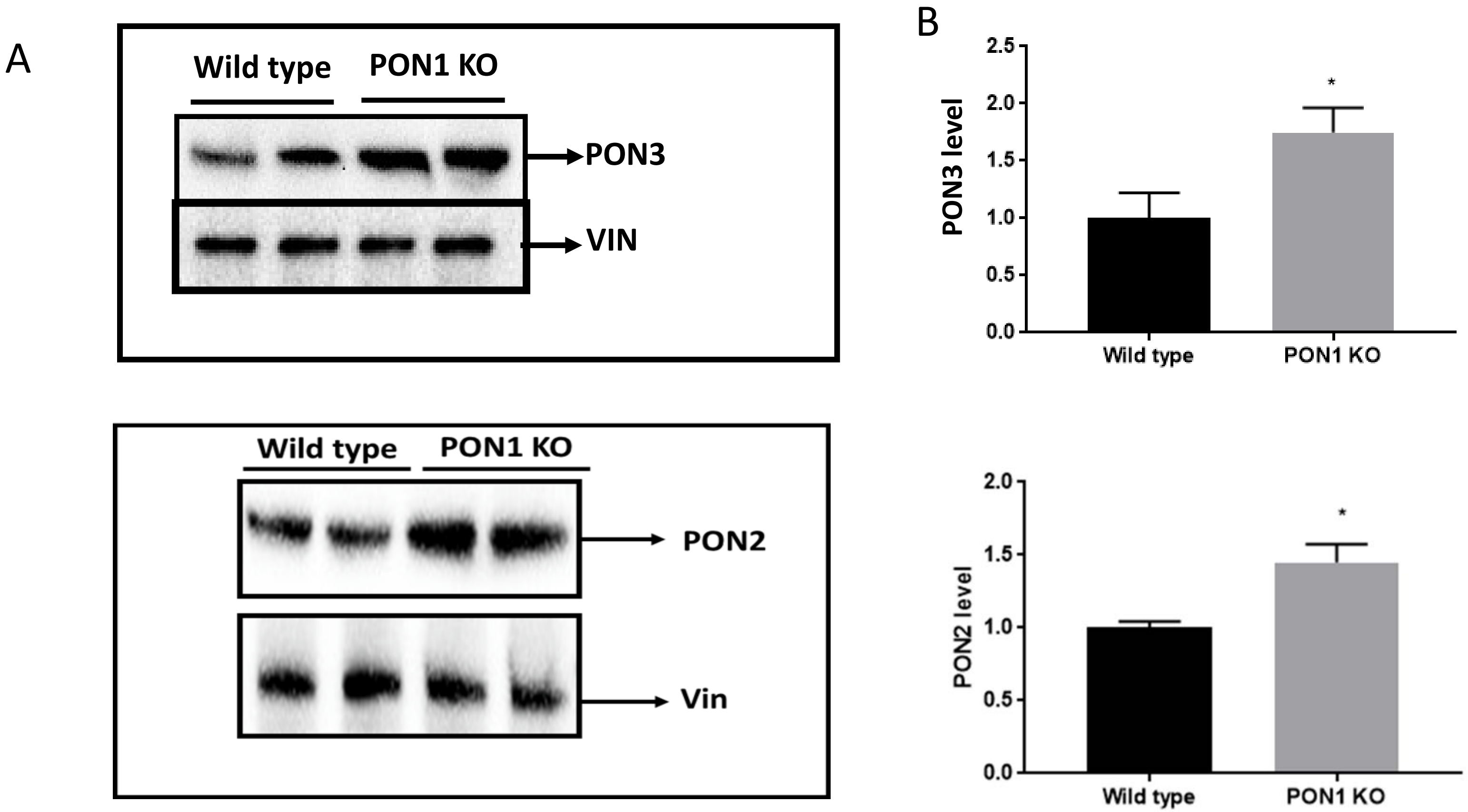

* P <0.05 compared to WT

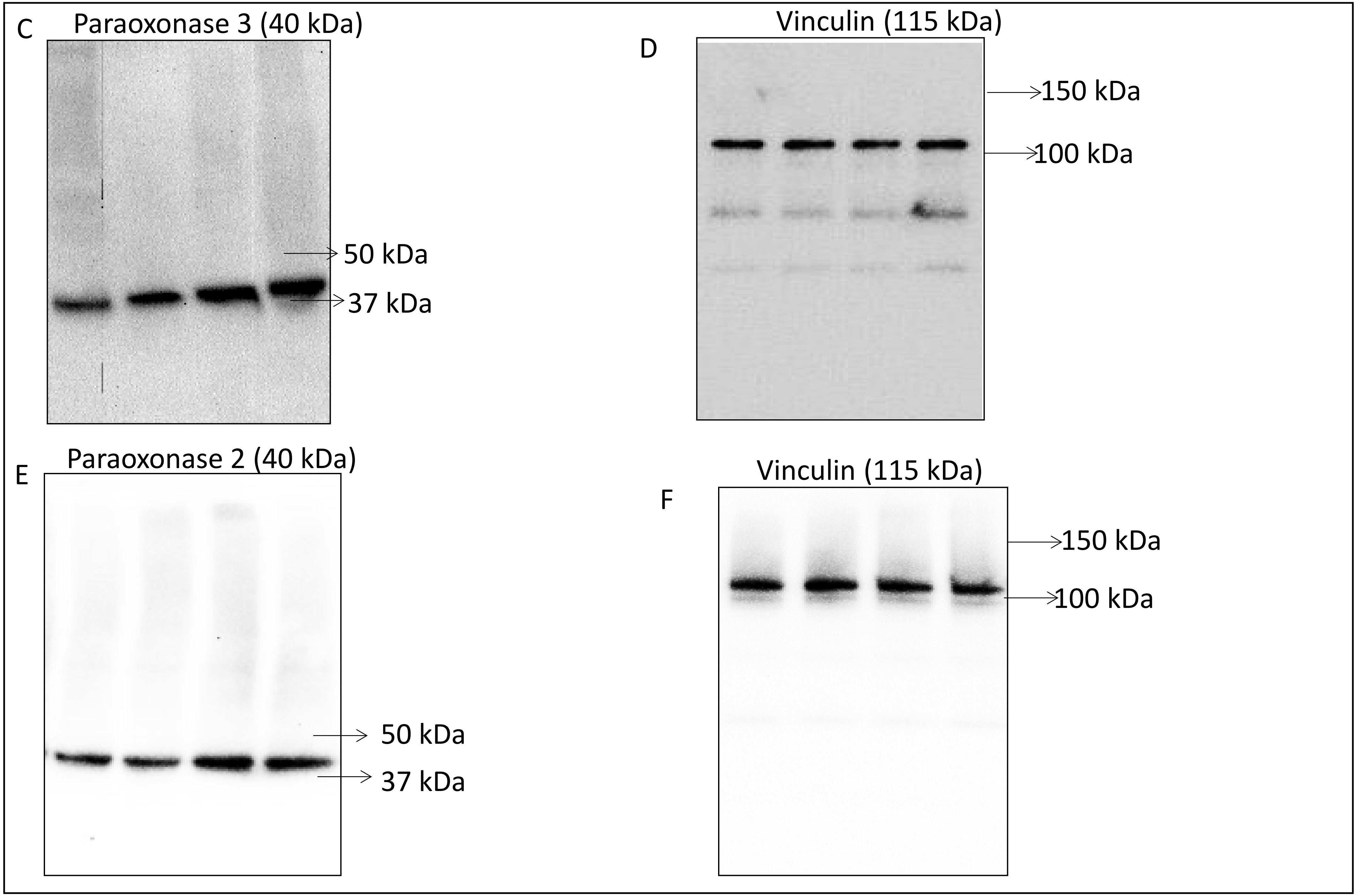
C
Paraoxonase 3 (40 kDa)
50 kDa
37 kDa
Vinculin (115 kDa)
D
150 kDa
100 kDa
E
Paraoxonase 2 (40 kDa)
50 kDa
37 kDa
Vinculin (115 kDa)
F
150 kDa
100 kDa

Supplementary Table 1: Primer Pairs used for Real-Time Reverse Transcription-PCR

Gene		Primer Sequence 5'-3'
GSTM1	Forward:	GCAGCTCATCATGCTCTGTT
	Reverse:	TTTTCTCAGGGATGGTCTTCA
GSTA2	Forward:	TGCCTGCCTTTGAAAAGGTGT
	Reverse:	AAGGCTGGCATCAAGCTCTTC
GSTM2	Forward:	CGCTTTGAGGGCCTGAAGAAGATA
	Reverse:	TAAGAGCTAGGGCCAGCAGAG
GSTM3	Forward:	GCCTGGATTTTCCCAATCTGC
	Reverse:	TCTCCAAAGTATCCACACGGA
GSTM6	Forward:	TGGTTTGCAGGGGACAAGAT
	Reverse:	TTCCTCAGGCCCTCAAAGC
GPX6	Forward:	CACATTGCAGGAGGAGCTGA
	Reverse:	GCCTGGACGCACATACTTGA
GAPDH	Forward:	TGAGGCCGGTGCTGAGTATGTCG
	Reverse:	CCACAGTCTTCTGGGTGGTG

Supplementary Table 2: Genes Differentially Associated with Arthritis Activity in K/BxN Mice

Gene ID	Fold Change	P value	Adjusted P value
Abcg1	14.97195	0.00004	0.05081
Arhgdib	22.47548	0.00004	0.05081
Atp11a	5.57669	0.00006	0.05081
C1qb	11.39285	0.00005	0.05081
Ccnd2	20.76950	0.00001	0.05081
Fcer1g	26.01673	0.00004	0.05081
Fcgr4	88.77787	0.00001	0.05081
Irf7	3.77039	0.00006	0.05081
Itgb2	51.48896	0.00004	0.05081
Laptm5	20.21389	0.00000	0.05081
Pld4	37.26168	0.00003	0.05081
Ptprc	31.74898	0.00002	0.05081
Tle1	-3.04678	0.00006	0.05081
Tmsb4x	15.24136	0.00003	0.05081
Cyp2a22	-4.29671	0.00008	0.05086
Plin5	-6.76273	0.00007	0.05086
Srebf1	5.65882	0.00008	0.05086
Ucp2	9.89334	0.00008	0.05086
4632428N05Rik	11.78662	0.00016	0.05266
Asb2	75.01430	0.00024	0.05266
Atp13a2	3.00608	0.00021	0.05266
C1qc	8.69364	0.00016	0.05266
Cd300ld	32.21202	0.00019	0.05266
Cdkn1a	-13.31875	0.00021	0.05266
Clic1	4.78329	0.00026	0.05266
Csf1r	17.33508	0.00014	0.05266
Cxcl14	29.18718	0.00018	0.05266
Cybb	23.87605	0.00016	0.05266
Cyth4	38.90193	0.00023	0.05266
Dusp10	5.17288	0.00024	0.05266
Emr4	46.25871	0.00022	0.05266
Hck	38.47641	0.00022	0.05266
Hic1	6.07135	0.00022	0.05266
Iqgap1	15.85066	0.00017	0.05266
Irgm2	3.40389	0.00021	0.05266
Klhdc7a	4.49119	0.00015	0.05266
Lhpp	-3.53249	0.00025	0.05266
Lrat	3.53765	0.00023	0.05266
Lyn	5.19388	0.00026	0.05266
Mnt	3.31750	0.00012	0.05266
Mob3a	5.59689	0.00016	0.05266
Msn	12.08807	0.00022	0.05266

Myo1f	24.65739	0.00024	0.05266
Ngfr	4.35964	0.00024	0.05266
Pkm	9.67482	0.00024	0.05266
Prex1	7.61331	0.00019	0.05266
Prps2	3.05312	0.00014	0.05266
Selplg	25.22007	0.00018	0.05266
Sirpa	13.98553	0.00013	0.05266
Slc11a1	21.64922	0.00016	0.05266
Sorl1	34.54970	0.00024	0.05266
Spi1	18.43188	0.00021	0.05266
Syk	26.02219	0.00017	0.05266
Themis2	43.38085	0.00022	0.05266
Tnfsf10	5.77717	0.00022	0.05266
Wee1	6.76254	0.00019	0.05266
Zfp516	3.78788	0.00015	0.05266
Slc37a2	17.77988	0.00027	0.05344
C1qa	7.51670	0.00028	0.05381
Plekho2	15.01799	0.00028	0.05381
Banp	-4.35110	0.00032	0.05413
Bmf	6.57341	0.00031	0.05413
Cd97	11.98734	0.00031	0.05413
Ezr	8.81776	0.00030	0.05413
Mbp	35.63784	0.00032	0.05413
Safb	-3.35721	0.00030	0.05413
Samhd1	6.77910	0.00031	0.05413
Tpd52	4.51639	0.00031	0.05413
Cd53	23.21007	0.00033	0.05542
Ctss	9.08932	0.00034	0.05598
Got1	-3.50098	0.00037	0.05612
Inf2	3.08688	0.00036	0.05612
Irf5	7.73829	0.00040	0.05612
Nlrp12	-4.21970	0.00038	0.05612
Pilra	28.53981	0.00035	0.05612
Pld1	3.11646	0.00040	0.05612
Rorc	3.62930	0.00038	0.05612
Scd2	6.03027	0.00037	0.05612
Slc25a22	-3.95047	0.00041	0.05612
Slc43a2	6.41105	0.00035	0.05612
Sult2a6	-165.27291	0.00038	0.05612
Zeb2	7.41143	0.00038	0.05612
Swap70	5.25019	0.00041	0.05612
Unc93b1	3.39946	0.00043	0.05760
Rac2	19.64857	0.00044	0.05784
Sdc3	9.45987	0.00044	0.05784
Tnfrsf14	10.54638	0.00046	0.05905

Hcls1	12.85134	0.00047	0.05908
Ly6e	3.07758	0.00046	0.05908
G6pdx	11.58602	0.00050	0.05953
Grem2	5.68589	0.00050	0.05953
Itgal	27.15682	0.00050	0.05953
Ms4a6c	14.44137	0.00049	0.05953
Ptprj	3.95871	0.00052	0.05953
Rassf5	3.18926	0.00052	0.05953
Wdfy4	23.19815	0.00051	0.05953
Ctsc	3.09276	0.00056	0.06266
Eml6	82.98544	0.00056	0.06266
Mapkapk3	7.69638	0.00055	0.06266
Tyrobp	12.14921	0.00056	0.06266
Fyb	13.31563	0.00057	0.06326
2310039H08Rik	-3.04507	0.00059	0.06357
Cd300e	141.26631	0.00058	0.06357
Pip4k2a	12.06037	0.00061	0.06357
Rgs2	10.70828	0.00061	0.06357
Slc25a34	-4.09599	0.00061	0.06357
Emr1	22.14801	0.00062	0.06385
Plin4	-7.23125	0.00062	0.06385
Sash3	28.71144	0.00063	0.06399
Fam129a	25.27438	0.00066	0.06414
Fmnl1	36.22694	0.00065	0.06414
Itga9	6.60679	0.00066	0.06414
Phf11b	34.95135	0.00064	0.06414
St8sia4	40.62112	0.00065	0.06414
Gsap	3.46288	0.00068	0.06473
Snn	9.33551	0.00070	0.06473
Ston2	3.94497	0.00068	0.06473
Tk1	3.23823	0.00068	0.06473
AB124611	12.86106	0.00072	0.06490
Clec4a3	47.49841	0.00072	0.06490
Coro1a	19.95353	0.00072	0.06490
Cyth3	9.78856	0.00075	0.06490
Fcgr1	14.62505	0.00076	0.06490
Folr2	8.59507	0.00073	0.06490
Lcp2	29.53764	0.00075	0.06490
Tln1	3.26809	0.00072	0.06490
Tmem82	-3.13460	0.00074	0.06490
Top2a	12.61896	0.00076	0.06490
Arl4c	9.74655	0.00079	0.06650
Wipf1	7.05382	0.00079	0.06650
Ptpn6	3.67467	0.00081	0.06735
Fxyd5	7.87133	0.00084	0.06768

Ckb	13.07818	0.00085	0.06841
Dclk3	3.05195	0.00089	0.06931
Phospho1	-5.80528	0.00089	0.06931
Sult2a4	-131.22596	0.00089	0.06931
Fam49b	5.99834	0.00091	0.07031
Flna	9.06952	0.00091	0.07031
Tnfaip2	4.13439	0.00092	0.07058
Evl	17.40789	0.00093	0.07085
1600002H07Rik	-3.45080	0.00095	0.07136
Bin2	17.36194	0.00097	0.07136
Eif4ebp3	-5.15250	0.00099	0.07136
I830012O16Rik	3.98598	0.00098	0.07136
Lrfn3	-3.15587	0.00096	0.07136
Pilrb1	118.86397	0.00097	0.07136
Ppm1h	8.39953	0.00099	0.07136
Pygb	5.74490	0.00099	0.07136
Clec12a	21.68960	0.00100	0.07156
Apex2	-3.78328	0.00101	0.07166
Ablim3	-3.01647	0.00103	0.07292
Rasa3	5.25115	0.00104	0.07292
Tbc1d1	6.33669	0.00104	0.07297
Brca2	3.19913	0.00105	0.07337
Apobr	18.28704	0.00109	0.07378
Axl	8.61888	0.00108	0.07378
Ccdc88b	16.39637	0.00111	0.07378
Cd5l	10.92174	0.00112	0.07378
Clec4f	3.97400	0.00108	0.07378
Cth	-3.17690	0.00108	0.07378
Fam117a	6.48236	0.00112	0.07378
Pla2g15	3.13165	0.00108	0.07378
Plin2	-3.72976	0.00112	0.07378
Pltp	6.56760	0.00113	0.07378
Cd300a	6.38887	0.00114	0.07383
Spn	52.17953	0.00114	0.07383
Acsl3	4.20942	0.00121	0.07447
Actg1	4.42489	0.00116	0.07447
Asl	-3.00147	0.00123	0.07447
Cd79b	26.16901	0.00117	0.07447
Chst2	6.73807	0.00119	0.07447
Cmtm7	10.31284	0.00119	0.07447
Gbp10	6.16800	0.00123	0.07447
Kbtbd11	25.81244	0.00121	0.07447
Nfam1	14.60865	0.00123	0.07447
Rgs10	10.85387	0.00122	0.07447
Tgfbr2	3.79455	0.00123	0.07447

Clec4a1	73.59963	0.00125	0.07464
Lamb3	-5.79360	0.00125	0.07464
Lbh	3.52748	0.00127	0.07519
Sco2	-5.98731	0.00126	0.07519
Cotl1	5.31121	0.00129	0.07568
3930402G23Rik	-27.02894	0.00131	0.07615
Pla2g12a	-3.08666	0.00133	0.07615
Plcg2	21.84479	0.00133	0.07615
Sptb	17.77251	0.00136	0.07740
Tifab	9.91923	0.00138	0.07788
Ano6	5.00974	0.00147	0.07850
Bhlhe40	3.08972	0.00147	0.07850
Gm11710	474.58898	0.00149	0.07850
Gm11711	474.58898	0.00149	0.07850
Lair1	19.18961	0.00148	0.07850
Lfng	11.45276	0.00146	0.07850
Rbl1	9.71605	0.00146	0.07850
Lcp1	3.55626	0.00156	0.07882
Sowahc	5.37644	0.00155	0.07882
1810055G02Rik	-3.77506	0.00158	0.07920
Limd2	3.85052	0.00158	0.07920
Dock2	17.15272	0.00160	0.07986
Apobec1	3.53690	0.00163	0.07990
Cd68	13.15127	0.00165	0.07990
Lpcat2	9.10005	0.00162	0.07990
Mpeg1	5.47182	0.00163	0.07990
Tifa	3.65685	0.00164	0.07990
Cd180	41.73947	0.00166	0.08008
Myo9a	9.72526	0.00168	0.08037
2010003K11Rik	-7.67773	0.00174	0.08078
Apbb1ip	18.24973	0.00178	0.08078
Atf5	-3.27885	0.00171	0.08078
Epb4.1l3	14.59174	0.00173	0.08078
Lgals3	13.30669	0.00173	0.08078
Sirpb1b	226.06604	0.00175	0.08078
Tmed5	-3.24887	0.00173	0.08078
Tnfrsf12a	-3.65775	0.00176	0.08078
Fes	9.29764	0.00180	0.08083
Itgam	34.00564	0.00179	0.08083
Lrrc25	24.72874	0.00181	0.08083
Akr1b10	13.29758	0.00182	0.08102
Gngt2	17.89130	0.00192	0.08230
Insig2	-3.76804	0.00194	0.08230
Mllt3	3.50795	0.00194	0.08230
Nr1d1	-5.88233	0.00194	0.08230

Plekha2	5.95938	0.00190	0.08230
Siglece	19.58295	0.00191	0.08230
Adamdec1	31.68485	0.00196	0.08259
Cd72	45.53050	0.00200	0.08282
Lnpep	7.14756	0.00198	0.08282
Pycard	43.24472	0.00198	0.08282
Vasp	4.20947	0.00200	0.08282
Ncoa7	8.63056	0.00204	0.08331
Negr1	80.39327	0.00203	0.08331
Aldh18a1	11.79760	0.00211	0.08428
Ccr5	9.59908	0.00207	0.08428
Naip2	5.10185	0.00209	0.08428
Nckap1l	15.59334	0.00208	0.08428
Syne3	3.20094	0.00211	0.08428
Adap1	55.94309	0.00217	0.08516
Atad5	6.11654	0.00224	0.08516
Itgax	93.06149	0.00219	0.08516
Mical1	17.86479	0.00222	0.08516
Plek	26.50022	0.00224	0.08516
Tagln2	4.52065	0.00219	0.08516
Tnfrsf21	4.45653	0.00220	0.08516
Zbtb4	3.32267	0.00217	0.08516
Slfn8	8.62099	0.00227	0.08609
Inpp5d	11.59578	0.00233	0.08675
Prkcb	10.13485	0.00232	0.08675
St6galnac4	7.88943	0.00233	0.08675
Stk17b	13.82644	0.00233	0.08675
Vsig4	9.80301	0.00234	0.08675
C630043F03Rik	-3.11088	0.00239	0.08704
Cnn2	6.71362	0.00240	0.08704
Dock10	19.84142	0.00239	0.08704
Kcna2	5.93869	0.00239	0.08704
Pirb	19.42316	0.00241	0.08704
Atp8a1	5.68349	0.00244	0.08734
Map3k3	3.07737	0.00245	0.08746
Tlr9	30.48599	0.00247	0.08776
6430548M08Rik	8.41122	0.00248	0.08782
Capn5	7.52584	0.00248	0.08782
Apoa1	-3.05394	0.00250	0.08801
Sik1	5.01193	0.00253	0.08846
Abi3	6.54462	0.00260	0.08993
Fcna	4.31650	0.00259	0.08993
Rab3il1	5.61399	0.00259	0.08993
Ptpn22	36.96771	0.00267	0.09084
Cd52	19.42930	0.00270	0.09089

Cd44	9.76030	0.00272	0.09156
Aldh3b1	5.60537	0.00275	0.09181
9930111J21Rik2	137.02309	0.00282	0.09239
Cxcl9	7.47488	0.00287	0.09239
Ehbp1l1	5.14969	0.00287	0.09239
Ehd4	3.83021	0.00287	0.09239
Elf4	10.97817	0.00285	0.09239
Heg1	8.22429	0.00282	0.09239
Pdcd1lg2	124.28412	0.00283	0.09239
Rbm43	3.61834	0.00279	0.09239
Rinl	6.07197	0.00293	0.09351
Dsg1c	7.31658	0.00297	0.09431
Cd86	8.16747	0.00309	0.09482
Esr1	5.78970	0.00301	0.09482
Fam78a	15.20826	0.00304	0.09482
Fdft1	3.98840	0.00309	0.09482
Gm4794	-26.50556	0.00300	0.09482
Igsf8	3.34740	0.00301	0.09482
Prss53	-3.69996	0.00303	0.09482
Rnf144a	4.85622	0.00308	0.09482
Slc30a10	-3.06029	0.00302	0.09482
Tmem229b	9.80778	0.00307	0.09482
Apba1	42.02999	0.00314	0.09563
Apol11b	191.23443	0.00320	0.09563
Etv5	5.54066	0.00315	0.09563
Gm13157	4.65148	0.00317	0.09563
Grap2	131.56067	0.00324	0.09563
Ifi204	6.60444	0.00327	0.09563
Klra2	32.88885	0.00322	0.09563
Ppfia4	7.16514	0.00317	0.09563
Rtp4	3.08538	0.00321	0.09563
AI467606	14.67610	0.00344	0.09636
Bag3	-3.27654	0.00338	0.09636
Bank1	9.63987	0.00337	0.09636
Camkk1	27.27182	0.00348	0.09636
Elovl3	-11.96817	0.00338	0.09636
Lepr	-6.16970	0.00350	0.09636
Ntrk1	-14.08933	0.00347	0.09636
Plxnb3	42.59796	0.00344	0.09636
Prdm1	10.59251	0.00343	0.09636
Ptplad2	11.64439	0.00343	0.09636
Snx20	26.70192	0.00348	0.09636
Tgfb1	6.91209	0.00345	0.09636
Mki67	19.24766	0.00352	0.09661
Ccl6	10.22734	0.00353	0.09673

Ly75	6.91967	0.00357	0.09717
Slc15a3	11.26886	0.00358	0.09717
Akna	7.89589	0.00362	0.09773
Endod1	3.91900	0.00366	0.09773
Hmgb2	4.20491	0.00368	0.09773
Kcnab2	18.63330	0.00368	0.09773
Apoa4	-3.17309	0.00375	0.09805
Snx10	3.30616	0.00374	0.09805
Abr	6.70066	0.00382	0.09882
Gm1966	8.51361	0.00382	0.09882
A730008H23Rik	3.84028	0.00387	0.09956

Supplementary Table 3: Clinical and Laboratory Characteristics of Patients with Rheumatoid Arthritis and Healthy Controls

	Control	RA	RA-Ath
Age (years)	63 ± 9	65 ± 7	68 ± 6
Sex (% female)	81	88	75
Ethnicity (% hispanic)	13	19	31
Race (% caucasian)	81	88	75
ESR (mm/hr)	10 ± 9#	13 ± 10#	44 ± 36*
HSCRP (mg/L)	1.5 ± 1.8#	1.8 ± 3.6#	19.5 ± 22.3*
Total Cholesterol (mg/dL)	184 ± 24	188 ± 33	164 ± 38
HDL Cholesterol (mg/dL)	62 ± 17#	85 ± 23#	58 ± 21*
LDL Cholesterol (mg/dL)	104 ± 23#	96 ± 34	79 ± 29*
Triglycerides (mg/dL)	112 ± 54	73 ± 27*#	153 ± 105

RA-Ath: Rheumatoid Arthritis with carotid plaque on carotid ultrasound. RA: Rheumatoid Arthritis without carotid plaque. *p<0.05 compared to control. #p<0.05 compared to RA-Ath.

Supplementary Table 4: Association of BLMs with Clinical and Histologic Scores in Arthritic Mice

	**Clinical Score**	**Histology**		
		**Inflammation**	**Cartilage Erosion**	**Bone erosion**
**BLMs**				
TXB2	-0.49#	0.43#	0.37	0.43#
6t12epiLTB4	0.33	0.15	0.21	0.31
LTB4	0.46*	0.59*	0.51*	0.68*
15d-D12,14 PGJ2	0.48*	0.61*	0.57*	0.69*
13 HODE	0.71*	0.74*	0.74*	0.79*
9 HODE	0.65*	0.75*	0.72*	0.79*
17S-HDHA	0.72*	0.64*	0.58*	0.62*
15 HETE	0.67*	0.70*	0.66*	0.72*
14S-HDHA	0.59*	0.39#	0.51*	0.47*
11 HETE	0.48*	0.72*	0.66*	0.73*
12 HETE	0.46*	0.22	0.37	0.32
5 HETE	0.35#	0.64*	0.51*	0.60*
5-oxoETE	0.68*	0.76*	0.71*	0.76*

* $p<0.05$, # $p\leq 0.1$.

Supplementary Table 5: Genes Differentially Regulated in PON1Tg Mice Compared to WT Mice following Arthritis Induction

Gene ID	Fold Change	P value	Adjusted P value
Ethe1	4.35995	0.00003	0.40209
Gstm1	3.34495	0.00007	0.43121
Gstm3	11.93905	0.00011	0.46774
Tmie	2.87885	0.00018	0.56027
Fam198a	5.43446	0.00030	0.70660
Gpcpd1	-3.91164	0.00036	0.70660
Mad2l2	2.35727	0.00052	0.70660
Ptplad1	-2.17633	0.00055	0.70660
Dhrs3	2.03693	0.00072	0.70660
Ldha	-2.23318	0.00080	0.70660
Psen2	2.18119	0.00083	0.70660
Chka	3.08320	0.00090	0.70660
Rn45s	-6.31166	0.00090	0.70660
Cdkn1c	2.38700	0.00091	0.70660
Nr0b2	4.55830	0.00096	0.70660
Gstm6	2.54077	0.00103	0.70660
Serpina3n	-2.09340	0.00106	0.70660
Cyp4a31	-3.61811	0.00115	0.70660
Arl10	-3.51879	0.00123	0.70660
Pnkd	2.26669	0.00124	0.70660
Slc13a2	6.15249	0.00132	0.72095
Slc22a5	-2.73990	0.00152	0.76335
Gm4724	5.48853	0.00184	0.79679
1500017E21Rik	5.10523	0.00208	0.84073
Cd300lg	2.09548	0.00228	0.84073
Tjp3	2.31474	0.00251	0.84073
Tmed8	-2.04537	0.00256	0.84073
Rasal2	-2.19521	0.00258	0.84073
Mcu	2.03322	0.00267	0.84073
Fhod1	2.13868	0.00303	0.84073
Hspa1b	-4.16829	0.00312	0.84073
Adck3	2.79351	0.00318	0.84073
Xpnpep3	-2.45631	0.00326	0.84073
Smim1	3.43390	0.00337	0.84073
Cpt1a	-2.19779	0.00346	0.84073
Chrna2	-2.93470	0.00357	0.84073
Fxyd3	7.58036	0.00361	0.84073
Tmem41a	-2.00465	0.00393	0.85496
Gstm2	2.42130	0.00395	0.85496
Chst14	3.68976	0.00406	0.85496

Lars2	-3.13979	0.00415	0.85496
Fam115a	3.00709	0.00434	0.85994
Ndrg1	3.36280	0.00487	0.85994
Aqp8	5.21491	0.00495	0.85994
Ninl	3.09157	0.00519	0.85994
Ptgr1	3.23449	0.00528	0.85994
Spry4	2.46440	0.00530	0.85994
Nupr1l	3.38105	0.00553	0.85994
Sel1l3	-2.72199	0.00598	0.85994
Mir6236	-2.57149	0.00616	0.85994
Rab30	-2.33600	0.00622	0.85994
Gck	3.79659	0.00631	0.85994
Tmem180	2.68998	0.00668	0.85994
Abca2	2.23721	0.00682	0.85994
St6galnac3	5.34922	0.00717	0.85994
Fam189b	3.76044	0.00719	0.85994
Fblim1	2.74404	0.00754	0.85994
Inhbe	-3.36994	0.00756	0.85994
Txnip	-2.37051	0.00765	0.85994
Zfp341	2.38427	0.00766	0.85994
Gas7	3.01086	0.00778	0.85994
Pkd2l2	2.23569	0.00800	0.85994
Ccne2	-3.51905	0.00805	0.85994
Slc10a2	-3.19282	0.00853	0.85994
Suv420h2	2.18004	0.00875	0.85994
Syne1	2.15667	0.00932	0.85994
Sox18	2.63855	0.00939	0.85994
Emcn	2.85013	0.00967	0.85994
Xrcc1	2.03290	0.00967	0.85994
St8sia3	-4.18529	0.00970	0.85994
Fam117a	2.19941	0.01045	0.85994
Fitm2	-2.54370	0.01059	0.85994
Cnpy4	2.06202	0.01064	0.85994
Nlrp12	-2.69191	0.01068	0.85994
BC039771	-3.44071	0.01069	0.85994
Praf2	2.45950	0.01086	0.85994
Ldb2	2.00786	0.01090	0.85994
Elovl2	-2.07563	0.01144	0.85994
Sprtn	-2.23847	0.01157	0.85994
BC021614	2.01544	0.01173	0.85994
Mvd	4.49786	0.01183	0.85994
Gsta2	3.20877	0.01204	0.85994
Mapk7	2.19505	0.01224	0.85994
Pcdhga12	-7.51584	0.01227	0.85994
Lipg	-2.24337	0.01243	0.85994

Limd2	2.03297	0.01251	0.85994
Shank2	5.49755	0.01304	0.85994
BC029214	2.99297	0.01306	0.85994
Pltp	2.08097	0.01353	0.85994
Hmgcs1	2.83176	0.01369	0.85994
Gm14305	-2.44225	0.01395	0.85994
Ces4a	-5.74275	0.01403	0.85994
Ten1	-2.86583	0.01475	0.85994
Cdca7	-2.58843	0.01505	0.85994
Ptms	2.00712	0.01569	0.85994
Nceh1	-2.34184	0.01611	0.85994
Smad9	-2.71021	0.01652	0.85994
Nes	2.97017	0.01666	0.85994
3110001I22Rik	-2.00724	0.01687	0.85994
Mid1	-2.09508	0.01691	0.85994
Dbn1	3.10081	0.01698	0.85994
Hmgn5	2.28545	0.01704	0.85994
Npas2	35.75054	0.01735	0.85994
Rwdd3	2.25358	0.01755	0.85994
Cbln3	2.50687	0.01757	0.85994
Smoc2	2.07046	0.01757	0.85994
Cd59b	3.34710	0.01796	0.85994
Ehd2	2.39730	0.01797	0.85994
Fbxl12os	-2.10366	0.01800	0.85994
Tbc1d30	-2.00980	0.01854	0.85994
Gm16845	2.82200	0.01859	0.85994
Trp53i11	2.13859	0.01906	0.85994
Nim1k	2.06705	0.01906	0.85994
Kcnip3	3.25354	0.01916	0.85994
Plac9b	2.56623	0.01920	0.85994
Tagln2	2.31062	0.01946	0.85994
Dcdc2a	2.18496	0.01986	0.85994
Retsat	-2.09362	0.01992	0.85994
Haghl	2.27329	0.02011	0.85994
Tmsb10	2.00767	0.02013	0.85994
Eif4ebp3	-3.12458	0.02038	0.85994
Ripply3	2.10594	0.02046	0.85994
Pex11a	-2.51433	0.02052	0.85994
Sh2d3c	2.00302	0.02061	0.85994
Prox1	-2.00449	0.02070	0.85994
Arhgap24	2.36853	0.02113	0.85994
Cnn2	2.28780	0.02159	0.85994
Dpt	2.69283	0.02162	0.85994
Samd10	2.60413	0.02213	0.85994
Lamb3	-3.90895	0.02227	0.85994

Mfsd7a	2.40335	0.02239	0.85994
Grk5	2.27212	0.02325	0.85994
Cd74	2.13561	0.02331	0.85994
2900008C10Rik	3.21433	0.02353	0.85994
Epor	3.27587	0.02353	0.85994
Paqr3	-3.01229	0.02364	0.85994
Gabra3	2.19379	0.02390	0.85994
Plp1	2.25366	0.02397	0.85994
Adat3	3.23634	0.02402	0.85994
Tmem88	2.34216	0.02443	0.85994
Cdk5r1	-3.72878	0.02457	0.85994
Hspa2	-2.25953	0.02463	0.85994
Ccdc151	2.96285	0.02467	0.85994
Mgst3	2.21430	0.02469	0.85994
Tdg	-9.56597	0.02471	0.85994
Pycard	2.75304	0.02517	0.85994
BC068157	3.15267	0.02550	0.85994
Crtap	2.01629	0.02550	0.85994
Arl1	-2.24864	0.02573	0.85994
Slc25a24	2.05912	0.02593	0.85994
Pla2r1	2.11542	0.02601	0.85994
Ndufa4l2	3.13046	0.02607	0.85994
2210404O09Rik	-2.67468	0.02615	0.85994
Apoa4	-4.21184	0.02621	0.85994
Zc2hc1a	3.44276	0.02628	0.85994
Gm12338	51.79957	0.02659	0.85994
Capn11	20.74722	0.02662	0.85994
Slc36a4	-3.87909	0.02724	0.85994
Lynx1	2.70053	0.02743	0.85994
Car15	4.88961	0.02759	0.85994
Kcna2	2.33743	0.02793	0.85994
Atxn7l2	2.50702	0.02837	0.85994
Pde3a	-5.04353	0.02858	0.85994
Loxl4	5.95203	0.02870	0.85994
Caprin2	2.61529	0.02871	0.85994
Fkbp10	4.35388	0.02881	0.85994
Cgref1	-12.36902	0.02888	0.85994
D030028A08Rik	-2.66457	0.02940	0.85994
Dact1	-2.01041	0.03015	0.85994
Nsun3	-2.10773	0.03023	0.85994
Slc41a3	-2.60313	0.03026	0.85994
Tert	2.83842	0.03034	0.85994
Zfp773	3.20205	0.03054	0.85994
Entpd7	-2.02298	0.03064	0.85994
4732471J01Rik	-2.86324	0.03069	0.85994

Tmem138	-2.01672	0.03073	0.85994
Cxcl9	2.55702	0.03074	0.85994
AI429214	-2.89895	0.03085	0.85994
Gucy1a2	2.34823	0.03195	0.85994
G0s2	-2.44481	0.03209	0.85994
Fgl1	-2.39701	0.03249	0.85994
Gstt3	2.45234	0.03261	0.85994
Gamt	2.13469	0.03263	0.85994
Tle2	3.04761	0.03264	0.85994
Lyrm7	-2.07301	0.03294	0.85994
Prtn3	-7.25757	0.03302	0.85994
Agap2	-2.10082	0.03320	0.85994
Psd	3.14639	0.03342	0.85994
Eif3j2	-2.15862	0.03356	0.85994
Adamts7	2.93271	0.03401	0.85994
Gm14308	4.83636	0.03406	0.85994
Taf4b	-2.05902	0.03458	0.85994
Pcyox1l	3.19619	0.03465	0.85994
Zdhhc1	2.00737	0.03474	0.85994
Hspa12b	2.18877	0.03542	0.85994
1700067K01Rik	2.59842	0.03550	0.85994
9430083A17Rik	-2.94558	0.03569	0.85994
A630007B06Rik	-2.00238	0.03572	0.85994
Capg	2.79868	0.03602	0.85994
Tonsl	3.19631	0.03622	0.85994
Gm12505	-2.06510	0.03658	0.85994
Tspan4	-2.19703	0.03671	0.85994
Adam11	2.13355	0.03790	0.85994
Kazn	2.89324	0.03807	0.85994
Hist1h1c	-2.10906	0.03830	0.85994
Cidec	-14.73152	0.03858	0.85994
Cyp4a10	-2.33389	0.03927	0.85994
Dysf	2.17710	0.03933	0.85994
Saa1	-6.70784	0.03982	0.85994
Spata22	2.06060	0.03982	0.85994
8430427H17Rik	2.04386	0.04004	0.85994
Cyp4a14	-2.91864	0.04027	0.85994
Dlgap1	2.54893	0.04043	0.85994
Wisp1	2.85642	0.04075	0.85994
Ptpre	2.08215	0.04101	0.85994
D930048N14Rik	2.44793	0.04149	0.85994
Ocstamp	-3.40056	0.04151	0.85994
Htra1	3.91607	0.04160	0.85994
Tram2	-2.42259	0.04178	0.85994
Mlxipl	2.30632	0.04180	0.85994

Kcnt2	-4.15047	0.04263	0.85994
Scarf2	2.00973	0.04354	0.85994
Exoc3l4	5.18935	0.04356	0.85994
Eif3j1	6.87007	0.04368	0.85994
Spsb1	2.09653	0.04385	0.85994
Tmem254c	32.35187	0.04392	0.85994
Saa2	-7.37119	0.04418	0.85994
Rbms3	2.16305	0.04430	0.85994
Oas1c	3.55080	0.04452	0.85994
Slc15a2	-9.56473	0.04467	0.85994
Gnat1	-2.07325	0.04675	0.85994
Gpx6	3.34126	0.04698	0.85994
Apcs	-2.10778	0.04744	0.85994
Mapk15	3.47288	0.04791	0.85994
Fam83f	2.38268	0.04879	0.85994
1810046K07Rik	2.70794	0.04884	0.85994
Ppfia4	2.51049	0.04959	0.85994
Slc17a4	2.27313	0.04986	0.85994

Supplementary Table 6: Correlation of Glutathione Pathway Gene Counts with Arthritis Activity and Bioactive Lipid Mediators

	Gpx6	Gsta2	Gstm1	Gstm2	Gstm3	Gstm6	Gstt3	Mgst3
**BLMs**								
**TXB2**	-0.02	0.05	0.02	0.20	-0.09	0.01	-0.22	-0.05
**6t12epiLTB4**	-0.06	-0.45#	-0.66*	-0.05	-0.41#	-0.70*	-0.10	-0.66*
**LTB4**	0.05	-0.68*	-0.70*	-0.47#	-0.49#	-0.60*	-0.41#	-0.61*
**15d-D12,14 PGJ2**	-0.16	-0.34	-0.39	-0.45#	-0.36	-0.43#	-0.28	-0.21
**13 HODE**	-0.54#	-0.35	-0.33	-0.28	-0.38	-0.25	-0.34	-0.31
**9 HODE**	-0.31	-0.62*	-0.69*	-0.58*	-0.61*	-0.64*	-0.58*	-0.61*
**17S-HDHA**	0.50#	-0.54#	-0.61*	-0.09	-0.45#	-0.59*	-0.04	-0.44
**15 HETE**	0.26	-0.31	-0.52#	0.14	-0.15	-0.56#	-0.22	-0.36
**14S-HDHA**	-0.03	-0.77*	-0.86*	-0.59*	-0.75*	-0.80*	-0.42#	-0.74*
**11 HETE**	-0.03	-0.59*	-0.70*	-0.29	-0.64*	-0.69*	-0.15	-0.48#
**12 HETE**	-0.23	-0.71*	-0.81*	-0.54*	-0.77*	-0.79*	-0.31	-0.73*
**5 HETE**	-0.11	-0.45#	-0.67*	-0.24	-0.59*	-0.69*	-0.20	-0.46#
**5-oxoETE**	-0.05	-0.56*	-0.60*	-0.53	-0.49#	-0.53*	-0.59*	-0.46#

#p ≤ 0.1 * p <0.05

Supplementary Table 7. Hepatic Transcriptome Analysis of PON1Tg Mice Compared to Control Mice Prior to Arthritis Induction

Kyoto Encyclopedia of Gnes and Genomes (KEGG) Pathway

ID	Genes	Fold Enrichment	P Value	Adjusted P value
mmu00900:Terpenoid backbone biosynthesis	MVD, HMGCR, FDPS, HMGCS1, MVK	37.95	0.0000061	0.0004
mmu03320:PPAR signaling pathway	CYP4A10, SCD1, EHHADH, CYP4A31, CYP4A14, PLTP	6.73	0.0058	0.1783
mmu00071:Fatty acid metabolism	CYP4A10, ALDH1B1, EHHADH, CYP4A31, CYP4A14	9.45	0.0080	0.1648
mmu00903:Limonene and pinene degradation	ALDH1B1, EHHADH, PNPLA3	21.25	0.0081	0.1279
mmu00100:Steroid biosynthesis	SQLE, NSDHL, FDFT1	18.75	0.0104	0.1309
mmu01040:Biosynthesis of unsaturated fatty acids	SCD1, ACOT2, ACOT1	11.81	0.0254	0.2493
mmu00650:Butanoate metabolism	ALDH1B1, EHHADH, HMGCS1	8.62	0.0454	0.3589
mmu00280:Valine, leucine and isoleucine degradation	ALDH1B1, EHHADH, HMGCS1	6.93	0.0669	0.4403
mmu00561:Glycerolipid metabolism	ALDH1B1, LIPG, PNPLA3	6.78	0.0695	0.4151
**mmu00480:Glutathione metabolism**	**GSTM1, GSTM2, GSTM3**	**6.13**	**0.0829**	**0.4398**
mmu00330:Arginine and proline metabolism	ALDH18A1, ALDH1B1, GAMT	6.01	0.0856	0.4202

Gene Ontology (GO) Molecular Function Pathway

ID	Genes	Fold Enrichment	P Value	Adjusted P Value
GO:0016765~transferase activity, transferring alkyl or aryl (other than methyl) groups	GSTM1, GSTM2, GSTM3, FDPS, MMAB, FDFT1	15.19	4.27E-05	0.0113
GO:0004091~carboxylesterase activity	CHKA, NCEH1, LIPG, ACOT2, ACOT1, PNPLA3	7.46	0.0012	0.1482
GO:0005520~insulin-like growth factor binding	WISP1, IGFBP2, IGFBP5	18.30	0.0113	0.6349
GO:0018685~alkane 1-monooxygenase activity	CYP4A10, CYP4A31, CYP4A14	134.22	0.0147	0.6264
**GO:0004364~glutathione transferase activity**	**GSTM1, GSTM2, GSTM3**	**15.49**	**0.0156**	**0.5666**
GO:0019838~growth factor binding	WISP1, NTRK2, IGFBP2, IGFBP5	7.46	0.0161	0.5124
GO:0016713~oxidoreductase activity, acting on paired donors, with incorporation or reduction of molecular oxygen, reduced iron-sulfur protein as one donor, and incorporation of one atom of oxygen	CYP4A10, CYP4A31, CYP4A14	53.69	0.0363	0.7550
GO:0016790~thiolester hydrolase activity	USP2, FASN, ACOT2, ACOT1	4.97	0.0456	0.7880
GO:0008889~glycerophosphodiester phosphodiesterase activity	GPCPD1, GDPD1	33.56	0.0575	0.8264
GO:0048037~cofactor binding	HMGCR, SQLE, HDC, EHHADH, FASN	2.97	0.0859	0.9082
GO:0008083~growth factor activity	NRG4, CNTF, INHBE, FGF12	3.81	0.0861	0.8867
GO:0020037~heme binding	CYP2G1, CYP4A10, CYP4A31, CYP26A1, CYP4A14	3.73	0.0904	0.8775
GO:0004659~prenyltransferase activity	FDPS, FDFT1	20.65	0.0918	0.8605
GO:0004806~triacylglycerol lipase activity	LIPG, PNPLA3	19.17	0.0985	0.8605

Gene Ontology (GO) Biological Process Pathway

ID	Genes	Fold Enrichment	P Value	Adjusted P Value
GO:0006695~cholesterol biosynthetic process	MVD, HMGCR, INSIG1, FDPS, HMGCS1, MVK, NSDHL, FDFT1	47.26	1.96E-10	1.79E-07
GO:0016126~sterol biosynthetic process	MVD, HMGCR, INSIG1, FDPS, HMGCS1, MVK, NSDHL, FDFT1	36.23	1.56E-09	7.15E-07
GO:0008203~cholesterol metabolic process	SREBF1, MVD, HMGCR, INSIG1, FDPS, HMGCS1, PCSK9, MVK, NSDHL, FDFT1	19.41	1.80E-09	5.50E-07
GO:0016125~sterol metabolic process	SREBF1, MVD, HMGCR, INSIG1, FDPS, HMGCS1, PCSK9, MVK, NSDHL, FDFT1	17.65	4.28E-09	9.81E-07
GO:0008299~isoprenoid biosynthetic process	MVD, HMGCR, FDPS, HMGCS1, MVK, FDFT1	37.06	4.43E-07	8.11E-05
GO:0006694~steroid biosynthetic process	MVD, HMGCR, INSIG1, FDPS, HMGCS1, MVK, NSDHL, FDFT1	15.31	7.99E-07	1.22E-04
GO:0008610~lipid biosynthetic process	SCD1, CHKA, MVD, HMGCR, HMGCS1, FDPS, PNPLA3, FDFT1, ST6GALNAC4, INSIG1, FASN, MVK, NSDHL	6.20	1.05E-06	1.37E-04
GO:0006720~isoprenoid metabolic process	MVD, HMGCR, FDPS, HMGCS1, CYP26A1, MVK, FDFT1	19.41	1.39E-06	1.60E-04
GO:0008202~steroid metabolic process	SREBF1, MVD, HMGCR, INSIG1, FDPS, HMGCS1, PCSK9, MVK, NSDHL, FDFT1	8.44	2.56E-06	2.61E-04
GO:0055114~oxidation reduction	CYP2G1, SCD1, PTGR1, ALDH18A1, HMGCR, EHHADH, CYP26A1, FDFT1, CYP4A10, SQLE, ALDH1B1, CYP4A31, FASN, LOXL4, CYP4A14, NSDHL	3.03	3.52E-04	0.031738
GO:0001558~regulation of cell growth	WISP1, NDN, TNFRSF12A, IGFBP2, CAPRIN2, IGFBP5	8.86	5.53E-04	0.044989
GO:0006637~acyl-CoA metabolic process	EHHADH, ACOT2, ACOT1	20.38	0.0092	0.5049
GO:0040008~regulation of growth	WISP1, NDN, TNFRSF12A, GAMT, IGFBP2, CAPRIN2, IGFBP5	3.72	0.0110	0.5414
GO:0035265~organ growth	LY6E, ADRA1B, GJE1	18.53	0.0110	0.5163
GO:0006575~cellular amino acid derivative metabolic process	CHKA, LY6E, HDC, GAMT, SLC22A5	4.82	0.0196	0.7012
GO:0006576~biogenic amine metabolic process	CHKA, LY6E, HDC, SLC22A5	6.79	0.0206	0.6958
GO:0010941~regulation of cell death	CNTF, ANK2, CD59B, KLF10, PYCARD, NLRP12, PCSK9, HSPA1A, HSPA1B, CIDEC, SPP1	2.41	0.0215	0.6894
GO:0006641~triglyceride metabolic process	INSIG1, PCSK9, PNPLA3	11.32	0.0282	0.7667
GO:0032535~regulation of cellular component size	FMN1, NDN, TNFRSF12A, ADRA1B, CAPRIN2	4.22	0.0300	0.7700
GO:0006639~acylglycerol metabolic process	INSIG1, PCSK9, PNPLA3	9.48	0.0391	0.8391
GO:0006662~glycerol ether metabolic process	INSIG1, PCSK9, PNPLA3	9.06	0.0425	0.8494
GO:0006638~neutral lipid metabolic process	INSIG1, PCSK9, PNPLA3	9.06	0.0425	0.8494
GO:0051186~cofactor metabolic process	HMGCR, EHHADH, ACOT2, ACOT1, MMAB	3.73	0.0439	0.8460
GO:0008361~regulation of cell size	NDN, TNFRSF12A, ADRA1B, CAPRIN2	5.03	0.0442	0.8351
GO:0006631~fatty acid metabolic process	CYP4A10, SCD1, EHHADH, CYP4A31, FASN, ACOT1	3.69	0.0454	0.8304
GO:0018904~organic ether metabolic process	INSIG1, PCSK9, PNPLA3	8.49	0.0477	0.8333
GO:0043281~regulation of caspase activity	PYCARD, NLRP12, HSPA1A, HSPA1B	8.15	0.0514	0.8439
GO:0052548~regulation of endopeptidase activity	PYCARD, NLRP12, HSPA1A, HSPA1B	8.15	0.0514	0.8439
GO:0043067~regulation of programmed cell death	CNTF, ANK2, KLF10, PYCARD, NLRP12, PCSK9, HSPA1A, HSPA1B, CIDEC, SPP1	2.18	0.0515	0.8338
GO:0040007~growth	CNTF, LY6E, NDN, ADRA1B, GJE1	3.52	0.0525	0.8285
GO:0052547~regulation of peptidase activity	PYCARD, NLRP12, HSPA1A, HSPA1B	7.99	0.0532	0.8221
GO:0060348~bone development	KLF10, INSIG1, IGFBP5, SPP1	4.61	0.0550	0.8220
GO:0042304~regulation of fatty acid biosynthetic process	SREBF1, INSIG1	30.20	0.0637	0.8570
GO:0046486~glycerolipid metabolic process	CHKA, INSIG1, PCSK9, PNPLA3	4.21	0.0680	0.8669
GO:0016042~lipid catabolic process	NCEH1, EHHADH, LIPG, PNPLA3	4.06	0.0744	0.8831
GO:0006732~coenzyme metabolic process	HMGCR, EHHADH, ACOT2, ACOT1	3.80	0.0865	0.9126
GO:0006766~vitamin metabolic process	CYP26A1, SLC22A5, MMAB	5.91	0.0901	0.9155
GO:0014073~response to tropane	ADRA1B, DLG4	20.90	0.0907	0.9111
GO:0042220~response to cocaine	ADRA1B, DLG4	20.90	0.0907	0.9111
GO:0007512~adult heart development	ADRA1B, SLC22A5	20.90	0.0907	0.9111

Supplementary Table 8: Glutathione Metabolism-related Gene Transcripts Correlate with PON1 Expression among HMDP Livers

Gene Number	Probeset ID	Gene Symbol	Gene Name	Correlation Coefficients (biweight midcorrelation)
329	1416531_at	Gsto1	glutathione S-transferase omega 1	0.401
486	1422072_a_at	Gstm6	glutathione S-transferase, mu 6	0.376
493	1423436_at	Gsta3	glutathione S-transferase, alpha 3	0.375
885	1418186_at	Gstt1	glutathione S-transferase, theta 1	0.336
